# The molecular pathogenesis of superoxide dismutase 1-linked ALS is promoted by low oxygen tension

**DOI:** 10.1007/s00401-019-01986-1

**Published:** 2019-03-12

**Authors:** Isil Keskin, Elin Forsgren, Manuela Lehmann, Peter M. Andersen, Thomas Brännström, Dale J. Lange, Matthis Synofzik, Ulrika Nordström, Per Zetterström, Stefan L. Marklund, Jonathan D. Gilthorpe

**Affiliations:** 1 Department of Medical Biosciences, Pathology, Umeå University, 90185 Umeå, Sweden; 2 Department of Pharmacology and Clinical Neuroscience, Umeå University, 90187 Umeå, Sweden; 3 Department of Neurology, Hospital for Special Surgery and Weill Cornell Medical Center, New York, NY 10021 USA; 4 Department of Neurodegenerative Diseases, Hertie Institute for Clinical Brain Research, University of Tübingen, Tübingen, Germany; 5 German Research Center for Neurodegenerative Diseases (DZNE), 72076 Tübingen, Germany; 6 Department of Medical Biosciences, Clinical Chemistry, Umeå University, 90185 Umeå, Sweden

**Keywords:** Amyotrophic lateral sclerosis (ALS), Superoxide dismutase 1 (SOD1), Disulfide bond, Oxygen tension, Protein disorder, Protein aggregation, Patient-derived cells

## Abstract

Mutations in *superoxide dismutase 1* (*SOD1*) cause amyotrophic lateral sclerosis (ALS). Disease pathogenesis is linked to destabilization, disorder and aggregation of the SOD1 protein. However, the non-genetic factors that promote disorder and the subsequent aggregation of SOD1 have not been studied. Mainly located to the reducing cytosol, mature SOD1 contains an oxidized disulfide bond that is important for its stability. Since O_2_ is required for formation of the bond, we reasoned that low O_2_ tension might be a risk factor for the pathological changes associated with ALS development. By combining biochemical approaches in an extensive range of genetically distinct patient-derived cell lines, we show that the disulfide bond is an Achilles heel of the SOD1 protein. Culture of patient-derived fibroblasts, astrocytes, and induced pluripotent stem cell-derived mixed motor neuron and astrocyte cultures (MNACs) under low O_2_ tensions caused reductive bond cleavage and increases in disordered SOD1. The effects were greatest in cells derived from patients carrying ALS-linked mutations in *SOD1*. However, significant increases also occurred in wild-type SOD1 in cultures derived from non-disease controls, and patients carrying mutations in other common ALS-linked genes. Compared to fibroblasts, MNACs showed far greater increases in SOD1 disorder and even aggregation of mutant SOD1s, in line with the vulnerability of the motor system to SOD1-mediated neurotoxicity. Our results show for the first time that O_2_ tension is a principal determinant of SOD1 stability in human patient-derived cells. Furthermore, we provide a mechanism by which non-genetic risk factors for ALS, such as aging and other conditions causing reduced vascular perfusion, could promote disease initiation and progression.

**Electronic supplementary material:**

The online version of this article (10.1007/s00401-019-01986-1) contains supplementary material, which is available to authorized users.

## Introduction

Amyotrophic lateral sclerosis (ALS) is characterized by adult-onset progressive degeneration of upper and lower motor neurons (MN). Usually, the disease begins focally and then spreads contiguously, resulting in progressive paralysis and death from respiratory failure [20]. Mutations in *superoxide dismutase 1* (*SOD1*) cause ALS [65], and are found in 1–9% of patients [3].

Human SOD1 is composed of two equal 153 amino acid-long subunits, each of which contains one copper and one zinc ion and a stabilizing C57–C146 disulfide bond required for dimerization. Of over 200 mutations identified in ALS patients, about 90% are missense and encode SOD1 variants that are destabilized to differing degrees. However, the proportion of disordered to natively folded protein varies widely between different mutations in patient cells [6, 47, 50, 51, 68, 71]. The other mutations encode mutants with structural alterations (insertions, fusions, internal deletions and C-terminal truncations). Most of the truncation mutations disrupt both the β-barrel core and the stabilizing intrasubunit C57–C146 disulfide bond of the protein. These mutants are intrinsically disordered, since they cannot fold correctly, and are rapidly degraded [13, 45, 50, 51, 68]. Based on these observations, it is likely that any common ALS-causing SOD1 species is disordered, lacks the C57–C146 disulfide bond, and is present at very low levels.

Although the involvement of wild-type SOD1 (SOD1^WT^) in ALS is contentious [9, 17, 27], there is an increasing body of evidence that has identified the presence of disordered and aggregated SOD1 in motor areas of the CNS in ALS patients that lack *SOD1* mutations [15, 31, 32, 33, 39, 56, 60, 62]. Co-culture of motor neurons with primary astrocytes derived from sporadic ALS patients supports the involvement of SOD1^WT^ in disease pathogenesis [41]. In addition, transgenic (Tg) mice that express human SOD1^WT^ to a sufficient level develop both SOD1 aggregation and a fatal ALS-like disease [40]. These findings suggest that disordered SOD1^WT^ could be more widely involved in ALS pathology than previously recognized.

SOD1 is an ancient enzyme and was secreted to the oxidizing extracellular space in early unicellular organisms, as well as in current α-bacteria [14, 58]. However, human SOD1 is localized primarily to the cytosol, which is strongly reducing. Hence, the C57–C146 bond could be an evolution-related Achilles heel of eukaryotic SOD1 due to vulnerability to reductive cleavage. The absence of the disulfide bond promotes disorder of the protein and its aggregation in vitro [21, 22, 35, 51, 53]. Moreover, soluble disordered and aggregated SOD1 in the CNS of human SOD1 Tg mice lacks the bond [10, 48, 77, 78]. Hence, C57–C146 appears to be a critical determinant of the role of SOD1 in ALS. However, factors that impact upon the formation and maintenance of the bond are not understood.

Age is the principal non-hereditary risk factor for ALS. It is associated with reduced perfusion in the CNS owing to vascular wall degeneration and neurovascular unit dysfunction [43]. Other suggested risk factors include smoking [37], CNS trauma, particularly to the motor cortex [66], embolisations of arteriovenous malformations [24, 54, 66, 72], transient ischemic attack, and stroke [72]. Strenuous physical activity has also been suggested to increase the risk for ALS [23], although this is contentious [38]. The underlying mechanisms are not understood, but a unifying characteristic could be reduced vascular perfusion leading to transient or chronic local CNS hypoxia.

The major pathway for C57–C146 disulfide formation is dependent on O_2_ and catalyzed by a copper chaperone for superoxide dismutase (CCS) [26, 30, 36]. Hence, we hypothesized that lowered O_2_ tension may impact negatively on SOD1 stability. To test this, we took advantage of an extensive collection of genetically defined cell lines derived from *SOD1* ALS and non-*SOD1* ALS patients, as well as from non-disease controls. As in vitro models of ALS, we used dermal fibroblasts, primary spinal cord-derived astrocytes, and induced pluripotent stem cell—(iPSC) derived mixed motor neuron and astrocyte cultures (MNACs). Analysis of lines carrying biochemically and structurally distinct SOD1 variants has enabled us to show, for the first time, that low O_2_ tension markedly increases the proportion of disordered, disulfide bond-reduced and aggregated SOD1 in a time and concentration-dependent manner and may be a risk factor for ALS development.

## Materials and methods

### Human materials

Samples from patients and non-disease controls (Supplementary Table 1), including blood samples for genotyping and skin biopsies for fibroblast culture, were collected with approval of the Regional Ethical Review Board in Umeå and in accordance with the principles of the Declaration of Helsinki (WMA, 1964), following written informed consent.

### Reagents and chemicals

Reagents and chemicals were obtained from Sigma or Thermo Fisher Scientific unless stated otherwise.

### *SOD1*, *FUS*, *TBK1* and *C9orf72* genotyping

ALS patients were diagnosed according to EFNS guidelines [4]. Blood was screened to identify *SOD1*, *C9orf72*, *TBK1* or *FUS* mutation carriers. Genotyping for *SOD1*, *C9orf72* [51] and *TBK1* [34] were performed as described. For *FUS,* only exons 2–6 and 11–15 were analyzed. All individuals tested negative for mutations in a panel of other ALS-linked genes (details available on request).

### Derivation of human fibroblasts

Fibroblasts were generated from a 3 mm punch skin biopsy (upper arm) from 10 ALS patients with mutations in *SOD1* (A4V, H46R, E78_R79insSI, N86S, D90A, G93A, L117V, D125Tfs*24 or G127Gfs*7 (G127X; two patients), one ALS patient with a mutation in *FUS* (Q23L), four ALS patients with mutations in *TBK1* (A417X, M598V, I450Kfs*14 or p.690-713del), one ALS and one FTD patient with massive intronic GGGGCC repeat-expansions in *C9orf72*, and four non-disease control individuals (Supplementary Table 1). All patients were heterozygous for their corresponding mutations except for the homozygous *SOD1*^*D90A*^ patient. All non-disease control subjects were relatives of ALS patients and tested negatively (wt/wt) for a panel of ALS-associated genes including *SOD1*, *C9orf72*, *TBK1*, *FUS* and *UBQLN2* (details available on request). The establishment of lines followed standard procedures [51].

### Generation and maintenance of iPSCs

Fibroblast lines were reprogrammed with either the mRNA Reprogramming Kit (Stemgent, Cambridge, MA, USA) [74] using a commercial service (Cellectis AB, Gothenburg, Sweden), or using episomal vectors [59] (Supplementary Table 1). After seeding at a density of 40,000 cells/cm^2^, iPSCs were cultured using the DEF-CS culture system (Takara Bio Europe, Gothenburg, Sweden). Media changes were performed every 24 h and cells were passaged using TrypLE every 3–4 days after reaching a density of 1.5–2 × 10^5^ cells/cm^2^.

### Generation of iPSC–MNACs

To differentiate MNACs, iPSCs at 90% confluence were switched into N2/B27 media, consisting of 1:1 DMEM/F12:Neurobasal, 1x Non Essential Amino Acids (NEAA; Millipore, Bedford, MA, USA), 2 mM l-glutamine, 1% (v/v) N2 supplement, 2% (v/v) B27 supplement and penicillin/streptomycin. Over the 14-day period of differentiation, N2/B27 media were supplemented with 1 μM all-*trans* retinoic acid (RA) and 1 μΜ smoothened agonist (SAG; Millipore, Bedford, MA, USA). For the first 6 days, cells were also subjected to dual SMAD inhibition with 10 μΜ SB431542 and 100 nM LDN (Stemgent, Lexington, MA, USA). On Day 7, dual SMAD inhibition was exchanged for 4 μΜ SU5402 (Stemgent, Lexington, MA, USA) and 5 μΜ DAPT (Selleck Chemicals, Houston, TX, USA) [52].

At Day 14, differentiated cells were dissociated with Accutase and plated onto poly-l-ornithine/laminin-coated 6-well plates (BD Biosciences, Franklin Lakes, NJ, USA) at a density of 100,000 cells/cm^2^, or 13 mm diameter coverslips (No. 1.5, VWR, Stockholm, Sweden) at 40,000 cells/cm^2^. MNAC cultures were maintained in MN culture media, consisting of Neurobasal, 1x NEAA, 2 mM l-glutamine, 1% (v/v) N2 supplement, 2% (v/v) B27 supplement, 0.4 mg/L ascorbic acid, 25 µM glutamate, 25 µM 2-mercaptoethanol, 1 μM RA, 20 µM Y-27632 (Abcam, Cambridge, UK) and penicillin/streptomycin. Cells were incubated in a humidified atmosphere at 37 °C supplemented with 5% (v/v) CO_2_. At days 11–13 days after plating, (days 24–26 after the onset of differentiation), MNACs were exposed to different O_2_ tensions prior to analysis.

### Generation of patient-derived primary astrocytes

A piece (2 cm) of the ventral horn from the thoracic spinal cord of a *SOD1*^*A4V*^ patient (Supplementary Table 1) was dissected at autopsy for cell isolation as described [41]. The tissue was diced and then dissociated using 2.5 U/ml papain and 0.5 U/ml Dispase (Stemcell Technology, Canada, Inc.) in 1xHBSS supplemented with 40 μg/ml DNAse at 37 °C for 45 min with mixing every 5 min. Cells were dissociated using a P1000 pipette tip and then mixed with KnockOut DMEM/F12 media containing 10% (v/v) foetal bovine serum (FBS), After filtering the cells through a 0.7 µm cell strainer, they were pelleted by centrifugation at 500*g* for 5 min. The cell pellet was resuspended in DMEM/F12 + 10% (v/v) FBS and mixed with an equal volume of Percoll (GE Healthcare, Chicago, Illinois). The mixture was centrifuged at 20,000*g* for 30 min at 4 °C and the flocculent layer above the red blood cell layer was collected, washed, pelleted and resuspended in primary cell media containing KnockOut DMEM/F12 + 10% (v/v) FBS supplemented with 20 ng/ml FGF-2, 20 ng/ml EGF, 20 ng/ml PDGF-AB (all from Peprotech, Rocky Hill, NJ), 2 mM GlutaMAX Supplement, 1x StemPro Neural Supplement and penicillin/streptomycin. The cells were cultured on CELLstart-coated plates and after 24 h the medium was replaced with serum-free primary cell media. Half of the medium was replaced every 2 days over 4–5 weeks until the cells reached 80% confluence. The cells were then passaged, expanded and plated either in coated 6-well plates (BD Biosciences, Franklin Lakes, NJ, USA), for exposure to different O_2_ tensions, or on 13 mm coated coverslips (No. 1.5, VWR, Stockholm, Sweden) for immunocytochemistry.

### Exposure to different oxygen tensions

Fibroblasts were plated at 8000–16,000 cells/cm^2^ in 6-well plates (BD Biosciences, Franklin Lakes, NJ, USA) and incubated in a humidified atmosphere at 37 °C with 5% (v/v) CO_2_. After reaching 75–85% confluence, the media were replaced prior to exposure to different O_2_ tensions. Differentiated MNACs were exposed to different O_2_ concentrations without a media change. For O_2_ tensions lower than 19%, cells were incubated in a sealed, humidified chamber (Biospherix, Lacona, NY, USA) gassed with gas mixtures (1–10% O_2_, 5% CO_2_, 94–85% N_2_) at 37 °C. The O_2_ concentration was maintained using a ProOx P110 oxygen controller (Biospherix, Lacona, NY, USA) supplied with N_2_ gas. For each experiment, cells were also cultured in parallel under humidified atmospheric O_2_ (19% O_2_, 5% CO_2_, 76% N_2_) in a standard tissue culture incubator.

### Immunocytochemistry

Cells were fixed in 3.8% (w/v) formaldehyde for 10 min at room temperature, and blocked with 10% (v/v) FBS in PBS containing 0.1% (v/v) Triton X-100 for 1 h at room temperature. Cells were incubated with primary antibodies overnight at 4 °C. The primary antibodies used were: anti-neuron-specific class III beta-tubulin (TUBB3, TUJ1, 1:7500, Covance Inc. Princeton, NJ, USA), microtubule-associated protein 2 (MAP2; 1:500, Millipore, Bedford, MA, USA), SMI32 (1:1000, Covance Inc. Princeton, NJ, USA), ISL1/2 (39.4D5, 1:5, developed by T.M. Jessell and S. Brenner-Morton was obtained from the Developmental Studies Hybridoma Bank, created by the NICHD of the NIH and maintained at The University of Iowa, Department of Biology, Iowa City, IA 52242), S100β (1:200) and vimentin (1:1000 Progen, Heidelberg, Germany). The following day, coverslips were washed and incubated with Alexa-Fluor conjugated secondary antibodies (1:1000) for 1 h at room temperature, and nuclei were counterstained with 4′,6-diamidino-2-phenylindole (DAPI; 0.3 µM). Cells were mounted in Aqua-Polymount (Polysciences, Inc., Warrington, PA, USA).

### In vitro cell cytotoxicity assays

Dead cells that had lost plasma membrane integrity were quantified using the luminogenic substrate AAF-Glo (CytoTox-Glo Cell Viability Assay, Promega, Madison, WI, USA) as previously described [51]. Cell viability was calculated by subtracting the luminescence signal obtained before permeabilization with digitonin, from the luminescence signal obtained afterward, according to the manufacturer’s protocol.

Cellular ATP content was determined using the CellTiter-Glo Luminescent Cell Viability Assay (Promega, Madison, WI, USA).

### Proteasome activity assay

A cell-based luminescent proteasome assay (Proteasome-Glo, Promega, Madison, WI, USA) was used to measure chymotrypsin-like proteasome activity as described [51]. The assay contains the luminogenic peptide substrate Suc-LLVY-aminoluciferin for determination of chymotrypsin-like activity of the proteasome.

### Cell extracts

Cells were washed with 37 °C PBS containing 40 mM iodoacetamide (IAM), which blocks reduced cysteine residues via alkylation to prevent oxidation of reduced C57–C146 disulfide bonds during sample processing [78]. The cells were detached with trypsin and then washed with PBS containing 40 mM IAM and 0.5% (v/v) FBS. After centrifugation of the suspension at 500*g* for 5 min, the cell pellet was collected and snap frozen on dry ice and stored in a − 80 °C freezer.

Cell pellets were thawed rapidly in a water bath at 25 °C for 1 min and then lysed in ice-cold PBS containing Complete EDTA-free protease inhibitor cocktail (Roche Diagnostics, Mannheim, Germany), 40 mM IAM and 0.5% (v/v) Nonidet P-40 (NP-40) using a Sonifier Cell Disrupter (Branson, Danbury, CT, USA). Lysates were centrifuged at 20,000*g* for 30 min at 4 °C and the supernatant was collected. The protein content of the cell lysate supernatant was determined using the BCA Protein Assay Kit. Disordered SOD1 was then analyzed immediately by misELISA (see below). Pellets were retained and snap frozen on dry ice and stored at − 80 °C for the subsequent analysis of SOD1 in detergent-insoluble aggregates (see below).

### Analysis of disordered and total SOD1 by ELISA

Disordered SOD1 was quantified in cell extracts using a specific ELISA (misELISA), described previously [76, 77]. The capture antibody was raised in rabbits against a peptide corresponding to aa 24–39 of the human SOD1 sequence. This antibody reacts only with highly disordered SOD1 species and lacks affinity for the natively folded protein [11, 31, 33]. A goat anti-human SOD1 antibody was used as a detection antibody. It was raised against SOD1 that had been denatured by incubation with guanidinium chloride and EDTA, and reacts preferentially with the disordered/unfolded protein [76]. For calibration of the misELISA, brain and spinal cord tissue from a Tg mouse expressing human SOD1^G127X^ was homogenized in 25 volumes 10 mM potassium phosphate buffer, pH 7.0 in 0.15 M NaCl, containing Complete Protease Inhibitor Cocktail (Roche Diagnostics, Mannheim, Germany) and 40 mM IAM. After centrifugation at 20,000*g* at 4 °C, the supernatant was divided into aliquots that were stored at − 80 °C. One unit of disordered SOD1 is defined as the amount present in 1 g wet weight of the original human SOD1^G127X^ standard.

The misELISA only detects disordered SOD1 species. There is no response to holoSOD1 or SOD1s that lack Cu and/or the C57–C146 disulfide bond, as long as the polypeptide is natively folded. Cell extracts are incubated for 1 h at 23 °C with the capture antibody. This temperature was selected as optimal because at the physiological 37 °C, some folded SOD1 species undergo continuous unfolding in highly diluted cell and tissue extracts, such as those analyzed here [77]. In addition, an increase in disorder at 37 °C compared to 23 °C is expected to show considerable variation between different SOD1s. For instance, the full-length SOD1^A4V^ and SOD1^G93A^ mutants show a significant degree of unfolding at 23 °C when Cu, Zn and the disulfide bond are removed (apo, disulfide-reduced SOD1). However, for SOD1^WT^ and stable mutants, the apo, disulfide-reduced forms melt at temperatures exceeding 40 °C [64]. Hence, more stable variants would show greater proportional increases in disorder at 37 °C than their unstable counterparts. SOD1^G127X^ and SOD1^D125Tfs*24^ C-terminal truncation mutants cannot fold natively and are intrinsically disordered. However, since they lack the C-terminal end, the truncation mutants are also expected to react less extensively with the detection antibody, which is raised against the entire denatured hSOD1 protein. Finally, mutations can also influence the conformation of immature SOD1 states, which could affect antibody reactivity [67, 70]. Hence, actual levels of disordered SOD1 at the physiological 37 °C are not mirrored equally for all SOD1 variants, and misELISA results are not directly comparable between cell lines expressing different SOD1s. However, for a given cell line expressing the same SOD1 variant, the effects of different O_2_ tensions on the levels of disordered SOD1 can be determined.

Following misELISA, cell extracts were snap frozen and subsequently analysed for total SOD1 with a sandwich ELISA based on rabbit capture and goat detection antibodies raised against native human SOD1 [76]. These antibodies bind to both native and disordered SOD1 with similar affinities.

### Design and analysis of misELISA experiments

All sets of independent experiments were conducted with cultures of each cell line grown in triplicate wells (i.e. three technical replicates) for each O_2_ tension. This was repeated on at least three occasions (i.e. three independent experiments). To analyze differences in absolute misELISA values, data from all replicates for one condition (19% or low O_2_) were pooled for statistical analyses (see below) (fibroblasts/primary astrocytes, Fig. 3a/b; MNACs, Fig. 4b). To compensate for variations in cell density and other methodological variations in sample preparation and analysis, we also analyzed the ratio of disordered SOD1 at 19% versus low O_2_. In this case, the mean of absolute misELISA values at 19% O_2_ was used to normalize ELISA values within all samples belonging to the same set (fibroblasts/primary astrocytes, Fig. 3c/d; MNACs, Fig. 4c), prior to statistical analyses (see below).

### Western blotting

Western blots were performed on Any kD or 18% (w/v) Criterion TGX precast gels (BioRad Laboratories, Hercules, CA, USA) as previously described [51]. Primary antibodies (see below) were incubated overnight at 4 °C. Horseradish peroxidase (HRP)-conjugated secondary antibodies (anti-mouse or anti-rabbit IgG; 1:10,000, Dako, Glostrup, Denmark) were incubated for 1 h at room temperature. ECL Select reagent (GE Healthcare Biosciences, Piscataway, NJ, USA) was used to detect the signal, which was recorded on a ChemiDoc Touch apparatus (BioRad Laboratories, Hercules, CA, USA) and analyzed using ImageLab software (BioRad Laboratories, Hercules, CA, USA).

The primary antibodies used for western blotting were; rabbit anti-human SOD1 antibodies raised against peptides corresponding to aa 24–39 (2.3 μg/ml), aa 57–72 (1.6 μg/ml), aa 144–153 (5.2 μg/ml) and aa 123–127 GQRWK (4.8 µg/ml, G127X-specific) [46], rabbit anti-CCS raised against peptides corresponding to aa 252–270 of the human CCS sequence (CCS, 0.9 μg/ml) [46], rabbit anti-CCS (1:1000 Santa Cruz Biotechnology, Dallas, TX, USA), rabbit anti-glutaredoxin-1 (1:250, Abcam, Cambridge, UK), mouse anti-β-actin (1:200,000; Millipore, Bedford, MA, USA) and rabbit anti-GAPDH (1:1000, Abcam, Cambridge, UK).

### Human SOD1 standards

For quantification by total SOD1 ELISA, or full-length SOD1 by western blotting, a human hemolysate was used with known SOD1 content calibrated against pure human SOD1, the concentration of which had been determined by quantitative amino acid analysis [57]. For quantification by misELISA, or SOD1^G127X^ by western blotting, an hSOD1^G127X^ Tg mouse extract was used in which the SOD1^G127X^ content had been determined by western blotting using the human SOD1-specific aa 24–39 antibody.

### Quantification of disulfide-reduced and -oxidized SOD1

The proportions of disulfide-reduced and -oxidized SOD1 were determined using western blotting (see above) following polyacrylamide gel electrophoresis under non-reducing conditions, i.e. by omitting reductant and adding 40 mM IAM to the sample buffer, as previously described [46, 77, 78].

### Immunocapture

The same rabbit anti-human SOD1 antibody raised against aa 24–39 of SOD1 used in the misELISA was cross-linked to Dynabeads^®^ M-270 Epoxy with the Dynabeads Antibody Coupling Kit. Beads were recovered with a magnet, washed with the supplied buffers to remove unbound antibody and equilibrated with PBS containing Complete EDTA-free protease inhibitor cocktail (Roche Diagnostics, Mannheim, Germany), 40 mM IAM and 0.5% (v/v) NP-40. Antibody-coated beads were incubated with 20,000*g* cell lysate supernatants (see above) for 1 h at 23 °C. Beads were washed five times with PBS containing the Complete EDTA-free protease inhibitor cocktail (Roche Diagnostics, Mannheim, Germany), 40 mM IAM and 0.5% (v/v) NP-40 and samples were eluted by boiling in 1x Sample Buffer containing 40 mM IAM. A proportion of the input and non-bound fractions (1/40th), as well as the entire immunocaptured fractions, were analyzed using non-reduced western blotting to determine the relative proportions of reduced and oxidized SOD1.

### Analysis of reduced and oxidized glutathione

GSH and GSSH were quantified by LC–MS [25]. After exposure of MNACs and fibroblasts to different O_2_ tensions (1–2–19% O_2_) for 24 h in 6-well plates, media were aspirated and 300 µL of ice-cold extraction mixture (0.25 µM glutathione (glycine-^13^C_2_,^15^N; GSH internal standard), 2.5% metaphosphoric acid, 1 mM EDTA and 0.1% formic acid) were added immediately to the cells. After scraping, the resulting cell suspensions were snap-frozen and stored at − 80 °C until analysis.

A tungsten bead was added to each tube and the frozen samples were homogenized immediately in a bead mill (Retsch MM400) at 30 oscillations/s for 1 min, followed by centrifugation at 22,000*g* for 20 min. The resulting supernatant (50 µL) was transferred to a LC–MS vial and 1 µL was injected and analyzed by LC–ESI–MSMS (1290 Infinity system from Agilent Technologies, with an Acquity UPLC HSS T3 column, thermostatted to 40 °C and coupled to an Agilent 6490 Triple quadrupole mass spectrometer). The analysis was performed essentially as described [18] by comparison to reduced glutathione (GSH) and oxidized glutathione (GSSG) solutions made in water (2.5 mM metaphosphoric acid, 1 mM EDTA, 0.1% formic acid) at twelve different calibration concentrations (0.01–10 µM) and containing the GSH internal standard.

GSH and GSSG concentrations were normalized to the protein content of the corresponding 22,000*g* pellets after extraction. Pellets were resuspended by sonication/boiling in 1x Sample Buffer. Protein estimation was performed using the BCA Protein Assay Kit and bovine serum albumin standards boiled in 1x Sample Buffer.

### Cycloheximide treatment

Fibroblasts were exposed to 19% O_2_ or 1% O_2_ as described above in the presence and absence of the protein synthesis inhibitor cycloheximide (CHX; 50 µg/ml) for 24 h, prior to harvest and analysis by misELISA and total SOD1 ELISA.

### Quantification of SOD1 in detergent-insoluble aggregates

Pellets were thawed on ice and washed twice by centrifugation at 20,000*g* for 30 min at 4 °C in cold PBS containing 0.5% (v/v) NP-40 to remove remaining detergent-soluble proteins. The material remaining in the pellet was used to determine the amount of detergent-insoluble/aggregated SOD1 by western blotting by comparison to a human SOD1 standard (see above). The amount of aggregated SOD1 in the detergent-insoluble fraction was expressed as a percentage of soluble SOD1 present in the corresponding cell extract, determined by total SOD1 ELISA.

### Statistical analyses

Statistical analyses were performed using Prism version 6.00 (GraphPad, La Jolla, CA, USA). To test for statistical significance between two groups, Mann–Whitney U test was used. For ratio analysis where data was normalized to the mean of 19% O_2_, the degree of freedom (*df*) was adjusted with *df* − 1. For statistical significance testing between multiple groups, Kruskal–Wallis test followed by Dunn’s post hoc test was used. Alpha ≤ 0.05 was used as the cut-off for significance. All values are given as mean ± SD.

## Results

### Low oxygen tension promotes SOD1 disorder in patient-derived cells

**Fig. 1:**
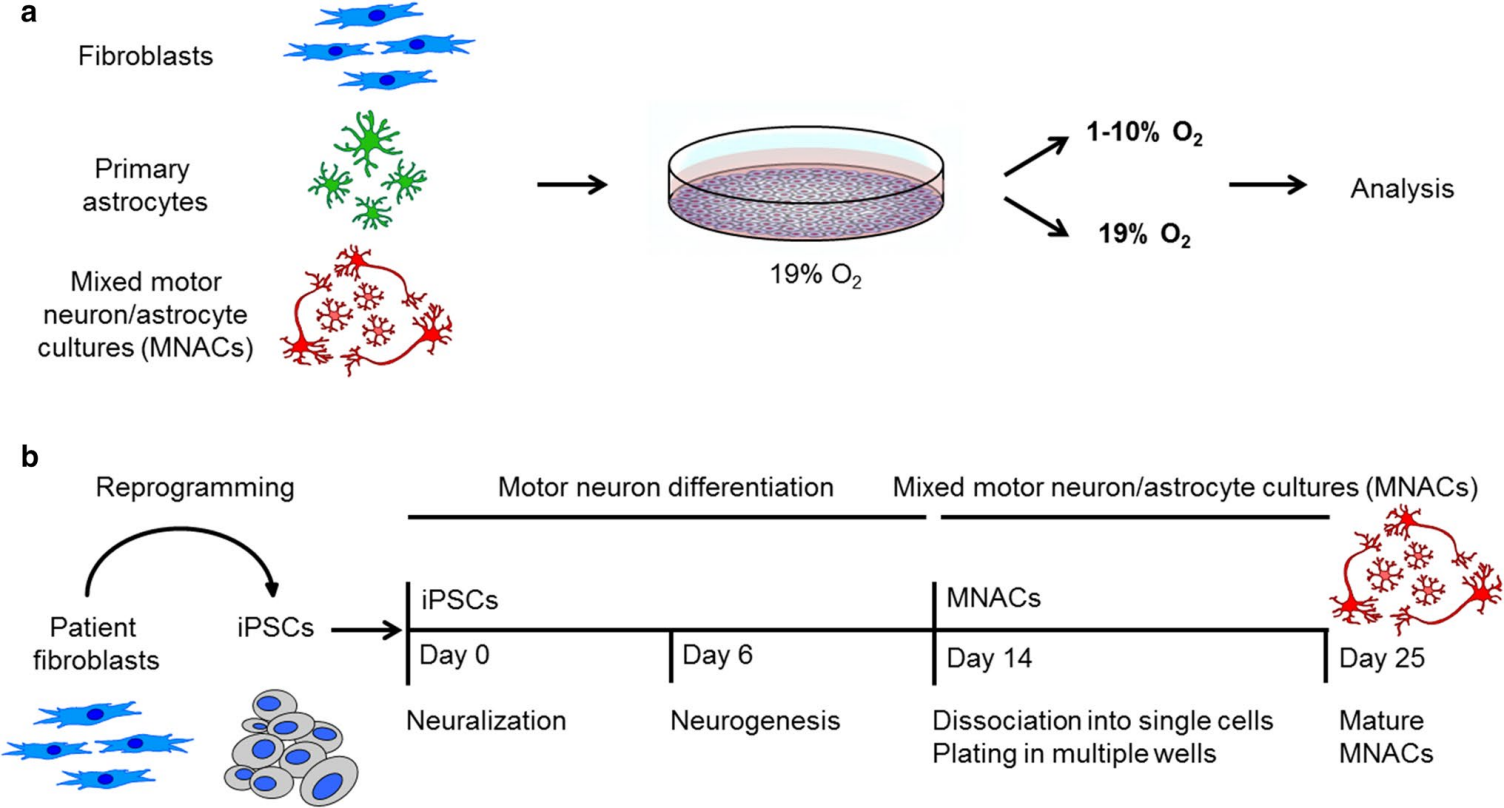
Experimental overview and generation of mixed motor neuron and astrocyte cultures (MNACs) derived from induced pluripotent stem cells (iPSCs). **a** Patient-derived fibroblasts, primary spinal cord ventral horn astrocytes and iPSC-derived MNACs were exposed to different O_2_ tensions for 24 h prior to analysis. **b** Patient-derived fibroblasts were reprogrammed to iPSCs using the four Yamanaka factors and then differentiated to a forelimb level, ventral spinal cord identity over 14 days. At Day 14, differentiated cells were dissociated to single cells and plated onto poly-l-ornithine/laminin-coated wells and matured for 10 more days. MNACs were used for O_2_ tension experiments at Day 25

Previously, we have used dermal fibroblasts from ALS patients as a cell culture model to study disordered SOD1 under physiological levels of SOD1 expression [51]. Cells cultured in vitro are typically maintained in humidified ambient air supplemented with 5% (v/v) CO_2_, resulting in an O_2_ tension of approximately 19%. However, under normal conditions, O_2_ tensions in the CNS are much lower, ranging between 0.2 and 5% [29, 55]. Moreover, various pathological conditions may lead to further reductions in local O_2_ tension. Therefore, we investigated the levels of disordered SOD1 in fibroblasts exposed to lowered O_2_ tensions for 24 h (Fig. 1a). We selected lines expressing a large number of different SOD1 variants with diverse molecular properties (Supplementary Table 1), to correlate these properties with potential increases in disordered SOD1, which we quantified with a specific ELISA (misELISA) [76].

**Fig. 2:**
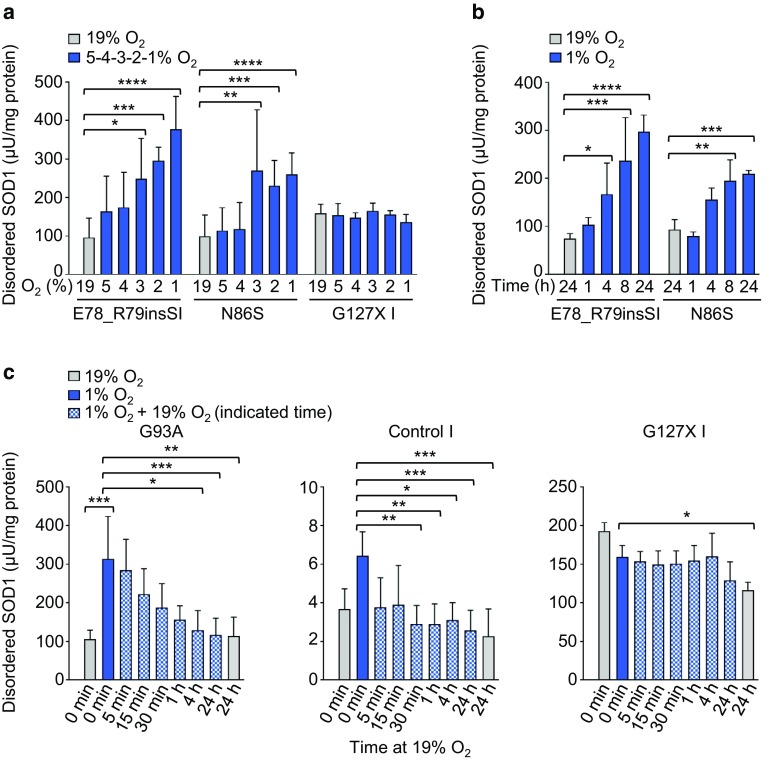
Concentration- and time-dependent appearance of disordered SOD1 in response to low O_2_ tensions. Disordered SOD1 was quantified in fibroblast extracts by misELISA and normalized to total protein. **a** O_2_ concentration—(1–2–3–4–5–19%) and **b** time—(1–4–8–24 h) dependent increases in disordered SOD1 in fibroblast lines. **c** Reversal of the response in disordered SOD1 following transfer of cultures from 1% O_2_ for 24 h to 19% O_2_ for the time indicated. **a**–**c** Data are expressed as the mean ± SD of nine technical replicates from three independent experiments, *p < 0.05, **p < 0.01, ***p < 0.001, ****p < 0.0001, analyzed by Kruskal–Wallis test followed by Dunn’s test

Lines carrying the *SOD1*^*N86S*^ and *SOD1*^*E78_R79insSI*^ mutations showed distinct O_2_ concentration-dependent responses in disordered SOD1. Increase in disordered SOD1 was detected from 3% but was most significant at 1% O_2_ (Fig. 2a). However, no increases in disordered SOD1 were found in a line carrying the *SOD1*^*G127X*^ mutation, which results in a C-terminally truncated protein that cannot fold properly due to a disrupted β-barrel and absence of the C57–C146 disulfide bond (Fig. 2a). Quantification of total SOD1 relative to total protein was performed to confirm that increases in disordered SOD1 were not due to increased SOD1 expression in response to low O_2_ tensions (Supplementary Fig. 1).

We next investigated the temporal response at 1% O_2_ and detected significant increases in disordered SOD1 at 4–8 h, which were sustained up to 24 h (Fig. 2b). To address whether the increase in disordered SOD1 was reversible, we examined different time points after returning cells from 24 h at 1% O_2_ to 19% O_2_. A rapid reversal was observed in the SOD1^G93A^ line with a half-life for disordered SOD1 of ~ 30 min, returning to baseline after 4 h (Fig. 2c). Reversal of the increase in disordered SOD1^WT^ in a control line was more rapid (Fig. 2c). No significant effects were seen in the SOD1^G127X^ line (Fig. 2c). Hence, we concluded that the disordered SOD1 response was inversely proportional to O_2_ tension and was also time-dependent, reversible, and appeared to require the C57–C146 disulfide bond. Amongst the conditions tested in fibroblasts, the most significant response observed was at 1% O_2_ for 24 h.

**Fig. 3:**
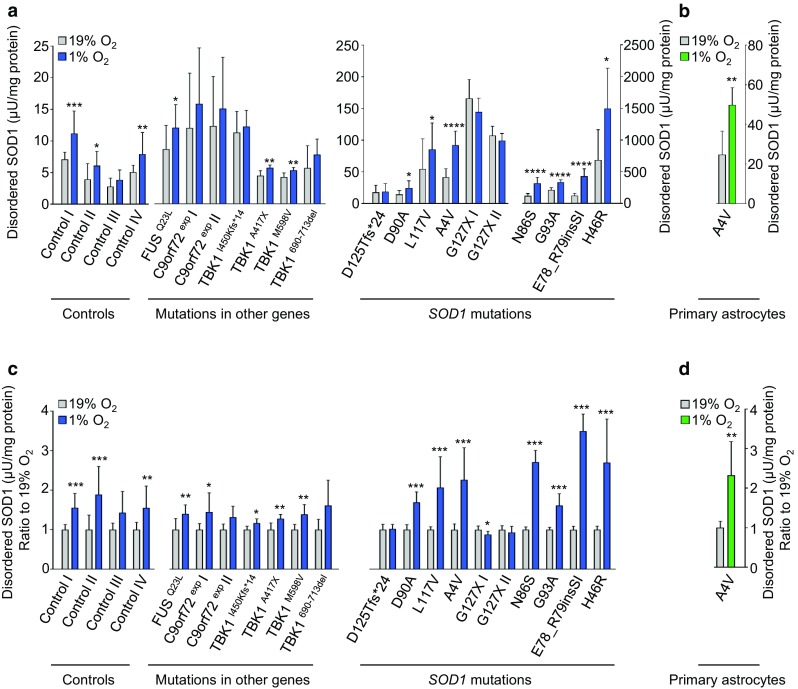
Response in disordered SOD1 to low oxygen tension in patient-derived cells. Following exposure to 1% O_2_ for 24 h, disordered SOD1 quantified in cell extracts by misELISA, normalized to total protein; **a** Patient-derived fibroblast lines, **b** Primary astrocytes. **c** and **d** Data presented as a ratio to the level present in replicate cultures maintained at 19% O_2_ for 24 h. **c** Patient-derived fibroblast lines, **d** Primary astrocytes. **a**–**d** Data are expressed as the mean ± SD of nine to 15 technical replicates from three to five independent experiments, *p < 0.05, **p < 0.01, ***p < 0.001, ****p < 0.0001, analyzed by Mann–Whitney U test. **c** and **d** The degree of freedom (*df*) was adjusted with *df* − 1. Blue bars = fibroblasts, green bars = primary astrocytes, grey bars = corresponding cell lines cultured at 19% O_2_

We proceeded to investigate the response in disordered SOD1 under the same conditions in a panel of non-disease control and patient-derived fibroblast lines (Supplementary Table 1). We also generated primary astrocytes from the ventral spinal cord of a *SOD1*^*A4V*^ ALS patient (Fig. 1a, Supplementary Fig. 2, and Supplementary Table 1). When comparing absolute levels of disordered SOD1 (Fig. 3a and b), or the ratio of disordered SOD1 at 1% versus 19% O_2_ (Fig. 3c and d), significant increases ranging from 1.6 to 3.5-fold were seen in all lines that carried full-length mutant SOD1s. Significant increases of lesser magnitude were also observed in most control lines, as well as in those from patients carrying ALS-linked *C9orf72*, *FUS* or *TBK1* mutations, which only contain SOD1^WT^. Analysis of disordered SOD1 ratios highlighted significant increases in all lines, apart from those carrying C-terminal SOD1 truncation mutations and the absence of C146: SOD1^*G127X*^ and *SOD1*^D125Tfs*2^. This supported the earlier conclusion that the response in disordered SOD1 to low O_2_ requires the C57–C146 disulfide bond.

### An enhanced response of disordered SOD1 in motor neuron/astrocyte cultures

Since ALS preferentially targets the motor system, we reprogrammed a subset of the patient-derived fibroblast lines to iPSCs and differentiated these to MNACs (Fig. 1b and Supplementary Table 1). Recently we have found that levels of disordered SOD1 are enhanced in MNACs compared to their corresponding fibroblasts, iPSCs and iPSC-derived sensory neurons  (unpublished data). After 10 days in culture post-differentiation (Day 25; Fig. 1b), MNACs contained ~ 5% neurons expressing tubulin beta-3 chain (TUBB3; Supplementary Fig. 3a and b) and ~ 95% astrocytes expressing astrocyte markers (unpublished data). A large proportion of neurons (78–84%) co-expressed the motor neuron markers ISL LIM homeobox 1/2 (ISL1/2) and non-phosphorylated neurofilament-H (SMI32), representing a limb innervating subtype that are highly susceptible to ALS [2].

**Fig. 4:**
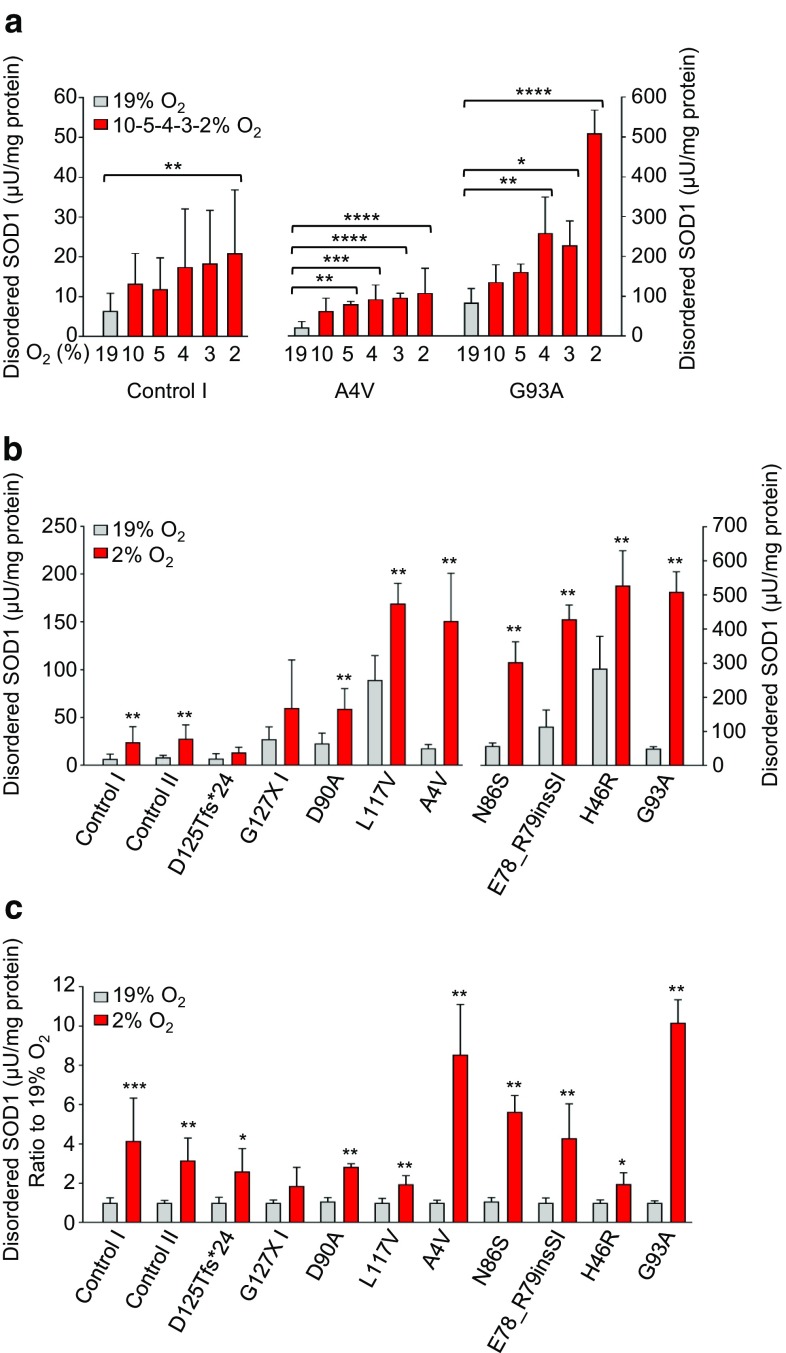
Response in disordered SOD1 to low oxygen tension is enhanced in patient-derived MNACs. Disordered SOD1 was quantified in MNAC extracts by misELISA and normalized to total protein. **a** O_2_ tension (2–3–4–5–10–19%)—dependent increases in disordered SOD1. Data are expressed as the mean ± SD of six technical replicates from two independent differentiations. **b** Disordered SOD1 was quantified in extracts by misELISA and normalized to total protein from a panel of MNACs following exposure to 2% O_2_ for 24 h. **c** Data presented as a ratio to the level present in replicate cultures maintained at 19% O_2_ for 24 h. **a**–**c** Data are expressed as the mean ± SD of six to 18 technical replicates from two to six independent differentiations, *p < 0.05, **p < 0.01, ***p < 0.001, analyzed by Mann–Whitney U test. **c** The degree of freedom (*df*) was adjusted with *df* − 1

We first titrated the dose–response in disordered SOD1 to O_2_ tension in MNACs from control, SOD1^A4V^ and SOD1^G93A^ lines. A significant increase in disordered SOD1 was detected at ≤ 4% and was maximal at 2% O_2_, which was enhanced in MNACs compared to fibroblasts in all three lines (Fig. 4a). Under the same conditions used for fibroblasts (1% O_2_ for 24 h), a significant degree of axonal fragmentation was observed (data not shown), indicative of neuronal stress. However, since axonal morphology (Supplementary Fig. 3b), cellular ATP levels (Supplementary Fig. 4a) and viability (Supplementary Fig. 4b) were not significantly affected by exposure to 2% O_2_ for 24 h, this was used to investigate the response in disordered SOD1 in MNACs.

Large increases in disordered SOD1, ranging up to tenfold, were observed in MNACs following exposure to 2% O_2_ for 24 h (Fig. 4b and c). The largest effects were found in several of the lines expressing full-length mutant SOD1s (E78_R79insSI, N86S, A4V, and G93A). However, in contrast to fibroblasts, a robust 3 to 4-fold increase was also seen in controls expressing SOD1^WT^ and in MNACs that were heterozygous for *SOD1*^*G127X*^ or *SOD1*^D125Tfs*24^ mutations and were present when comparing either absolute levels of disordered SOD1 (Fig. 4b) or the ratio (Fig. 4c) of disordered SOD1 within lines at 2% versus 19% O_2_. The increases in disordered SOD1 in MNACs carrying *SOD1* C-terminal truncation mutations are likely to represent SOD1^WT^, since the level of mutant protein did not increase in the SOD1^G127X^ line (Supplementary Fig. 5b).

Our previous study of patient-derived fibroblasts showed that disordered SOD1 is primarily degraded by the proteasome [51]. However, exposure to low O_2_ tension did not lead to a reduction in proteasome activity in MNAC extracts (Supplementary Fig. 6). Nor were there increases in SOD1^G127X^ protein in either soluble (Supplementary Fig. 5) or detergent-insoluble (Supplementary Fig. 11b) fractions, which both increased greatly upon proteasome inhibition [51]. Inhibition of the proteasome also leads to copious aggregation of SOD1^D125Tfs*24^ in fibroblasts [51], whereas no changes were found here in response to low O_2_ tension (see below; Fig. 8 and Supplementary Fig. 11a). Hence, increases in disordered SOD1 at low O_2_ tensions could not be attributed to a reduction in proteasome activity.

### Low oxygen tension promotes reductive cleavage of the disulfide bond

**Fig. 5:**
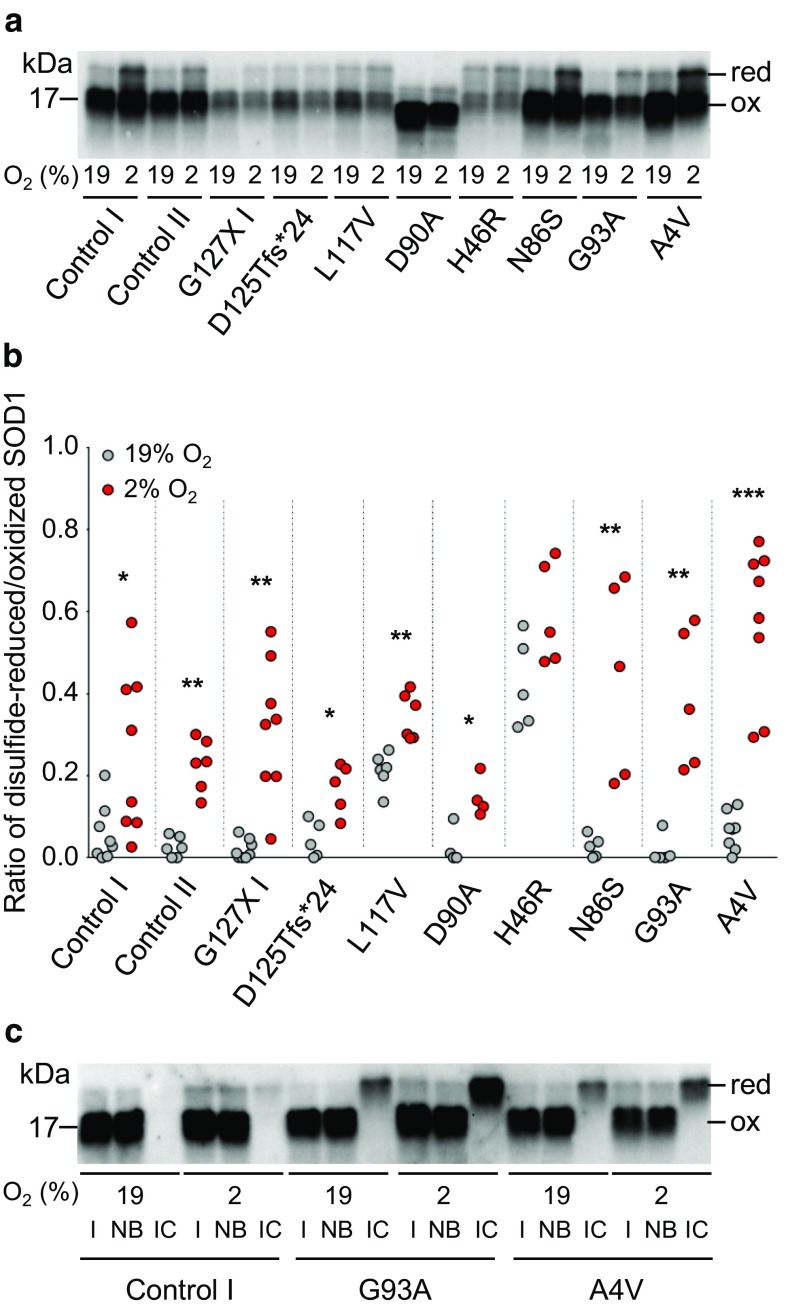
Low O_2_ tension promotes disulfide bond reduction and disorder of SOD1. Analysis of MNAC extracts following exposure to 19% or 2% O_2_ for 24 h. **a** Non-reducing SDS-PAGE followed by western blotting with an anti-SOD1 aa 24–39 antibody was used to resolve disulfide-reduced (red) and -oxidized (ox) SOD1. **b** Scatter plot showing the ratios of disulfide-reduced to -oxidized SOD1 at 19% (grey circles) versus 2% (red circles) O_2_. Data are expressed as the mean ± SD of four to eight technical replicates from two to six independent differentiations, *p < 0.05, **p < 0.01, ***p < 0.001, analyzed by Mann–Whitney U test comparing 19% versus 2% O_2_. **c** Western blot showing analysis of immunocaptured disordered SOD1. Input (I, 1/40th of the sample), non-bound (NB, 1/40th) and immunocaptured (IC, entire sample) fractions of SOD1 from MNACs cultured at either 19% O_2_, or 2% O_2_, for 24 h. ICs were performed using a rabbit anti-SOD1 aa 24–39 antibody immobilized on Dynabeads. Disulfide-reduced and -oxidized SOD1 were quantified using a rabbit anti-SOD1 aa 57–72 antibody. Only disulfide-reduced SOD1 was captured

We have shown that reduction of the C57–C146 bond is a prerequisite for the formation of disordered SOD1 in the CNS [77, 78]. To examine the status of the disulfide bond in MNACs we determined the proportions of disulfide-reduced and oxidized SOD1 by non-reduced western blotting (Fig. 5a and b). Culture at 2% O_2_ for 24 h resulted in significant increases in disulfide-reduced SOD1, both in control lines expressing SOD1^WT^ and those expressing mutant SOD1s. However, this was not the case for the SOD1^H46R^ line, where a large proportion of the mutant protein is known to be disulfide-reduced under control conditions [75].

### Disordered SOD1 in MNACs lacks the disulfide bond

To confirm that disordered SOD1 lacks the C57–C146 disulfide bond, we immunocaptured SOD1 from extracts of MNACs, which had been cultured at 19% or 2% O_2_, using the same antibody (SOD1 aa 24–39) and capture conditions (1 h at 23 °C) used in the misELISA. This antibody reacts with disordered SOD1, independent of the C57–C146 disulfide status [33]. Disulfide-reduced and -oxidized SOD1 were quantified by non-reduced western blotting (Fig. 5c). Input samples from control, SOD1^G93A^ and SOD1^A4V^ MNACs contained a majority of oxidized and a small proportion of reduced SOD1, which increased under low O_2_ conditions. Notably, an antibody specific for disordered SOD1 captured only disulfide-reduced SOD1. In control MNACs, disordered SOD1 was not detected at 19% O_2_ but increased at 2% O_2_ to ~ 1.4% of the disulfide-reduced SOD1 in the input sample. In SOD1^G93A^ and SOD1^A4V^ MNACs, disordered, disulfide-reduced SOD1 increased substantially (to 21% and 10% of the input samples at 2% O_2_, respectively). Hence, even in MNACs carrying mutant SOD1s, the majority of disulfide-reduced SOD1 retained an ordered structure. This agrees with observations in the spinal cord of SOD1 Tg mouse models, where approximately 5–10% of disulfide-reduced human SOD1 is disordered [77, 78].

### Low oxygen tension does not alter other determinants of SOD1 redox status

**Fig. 6:**
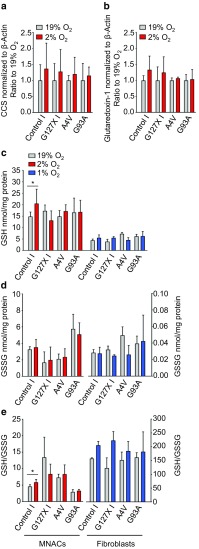
Low O_2_ tension does not alter known determinants of SOD1 C57–C146 redox status. Quantification of: **a** CCS or **b** glutaredoxin-1 in MNAC extracts by western blotting (Supplementary Fig. 7b), normalized to β-actin as a loading control. Cells were exposed to 19% O_2_ (grey bars) or 2% O_2_ (red bars) for 24 h. Data were plotted as a ratio of the level present in replicate cultures maintained at 19% O_2_ for 24 h and expressed as the mean ± SD of four to ten technical replicates from two to six independent differentiations, analyzed by Mann–Whitney U test. **c** GSH levels, **d** GSSG levels and **e** GSH/GSSG ratios determined in MNACs (left, red bars) and fibroblasts (right, blue bars). Data are expressed as the mean ± SD of six technical replicates from two independent differentiations for MNACs and of three technical replicates from one experiment for fibroblasts, *p < 0.05, analyzed by Mann–Whitney U test comparing 19% versus 2% O_2_ for MNACs

The main mechanism for oxidation of the C57–C146 disulfide bond involves catalysis by CCS and is dependent on O_2_ [26, 30, 36]. The bond can be reduced by a mechanism involving reducing equivalents from GSH via glutaredoxin-1 [19]. To determine whether levels of CCS or glutaredoxin-1 were affected by low O_2_ tension and thus responsible for increased disordered SOD1, we analyzed their levels in MNACs grown at 19% versus 2% O_2_. However, no significant changes were detected in CCS or glutaredoxin-1 levels by western blotting (Fig. 6a and b and Supplementary Fig. 7). Next, we quantified GSH and GSSG, which form the principal redox couple in the cytosol. GSH and GSSG concentrations were higher in MNACs than in fibroblasts, but exposure to low O_2_ tension did not lead to significant changes in the levels of GSH (Fig. 6c) or GSSG (Fig. 6d). The concentrations of GSSG were remarkably high in MNACs, resulting in very low GSH/GSSG ratios, but these were not affected by O_2_ tension (Fig. 6e). Hence, low O_2_ tensions do not induce disordered, disulfide-reduced SOD1 via gross perturbation of the GSH/GSSG redox couple [69].

### Maintenance of the disulfide bond and SOD1 structure is oxygen dependent

**Fig. 7:**
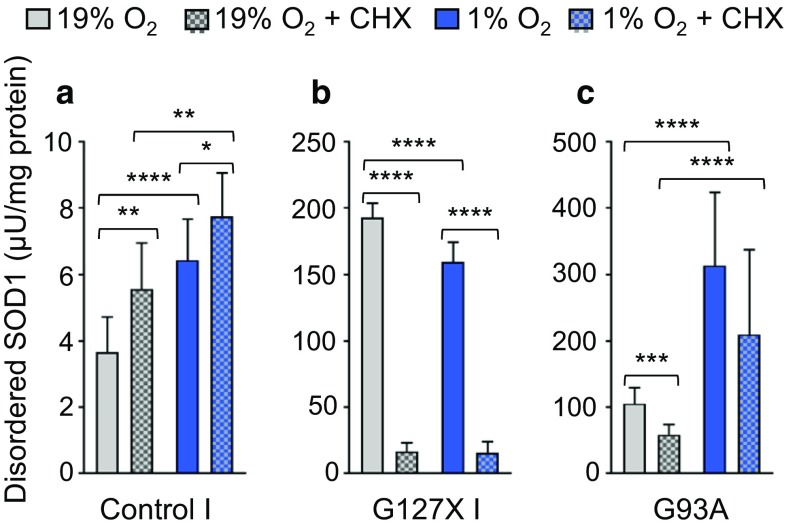
Low O_2_ tension increases disorder in both nascent and mature SOD1. Disordered SOD1 was quantified in fibroblast extracts by misELISA and normalized to total protein; **a** Control I, **b** SOD1^G127X^ and **c** SOD1^G93A^. Cells were grown at 19% O_2_ (grey bars) or 1% O_2_ (blue bars) for 24 h in the absence, or presence (checkerboard pattern) of cycloheximide (CHX; 50 µg/ml). Data are expressed as the mean ± SD of nine technical replicates from three independent experiments, *p < 0.05, **p < 0.01, ****p < 0.0001, analyzed by Mann–Whitney U test

Increase in disordered SOD1 at low O_2_ tension could be due to impaired disulfide oxidation of newly synthesized SOD1, or to the reduction of the disulfide bond in the mature protein. To distinguish the relative contribution of these two possible mechanisms, we compared the response in disordered SOD1 in fibroblasts cultured in the absence or presence of the protein synthesis inhibitor CHX for 24 h. No significant effects were seen on the expression of SOD1 relative to total protein following CHX treatment (Supplementary Fig. 8). Inhibition of protein synthesis led to a small increase in the level of disordered SOD1^WT^ in the control fibroblasts cultured at 19% (Fig. 7a). This suggests that CHX treatment had a slight effect on the capacity of the cells to handle disordered SOD1. However, it did not suppress the increase in disordered SOD1^WT^ in control fibroblasts at 1% O_2_. CHX also resulted in a striking reduction in disordered SOD1 in the SOD1^G127X^ line at both O_2_ tensions (Fig. 7b). This confirmed that the inhibition of protein synthesis was efficient. It also showed that CHX treatment did not significantly affect the degradation of the disordered mutant protein. In the SOD1^G93A^ line, CHX treatment resulted in a reduction in disordered SOD1 at both O_2_ tensions (Fig. 7c). Hence, a proportion of disordered SOD1 resulted from a lack of disulfide oxidation in newly synthesized SOD1^G93A^. However, the response in disordered SOD1 was still greatly enhanced at 1% compared to 19% O_2_ when protein synthesis was inhibited. Thus, O_2_ is also required for maintenance of the disulfide bond and structure of the existing pool of mature SOD1.

### Low oxygen tension promotes SOD1 aggregation

Since disulfide reduction and disorder promote SOD1 aggregation [21, 35, 53], we quantified the amount of SOD1 in detergent-resistant aggregates by western blotting. To identify the optimal antibody for this purpose, we compared antibodies to epitopes in the N-terminus (aa 24–39), the central region (aa 57–72) and the C-terminus (aa 144–153). A large number of apparently non-specific cross-reacting bands of unknown identities were evident (Supplementary Fig. 9). Since these bands differed between antibodies, we only quantified full-length SOD1 co-migrating with the SOD1 standard used for quantification. All three antibodies detected this band similarly. However, we used the aa 57–72 antibody because it was the most sensitive. For SOD1^G127X^ quantification, we used the aa 123–127 GQRWK antibody, which is specific for this mutant.

**Fig. 8:**
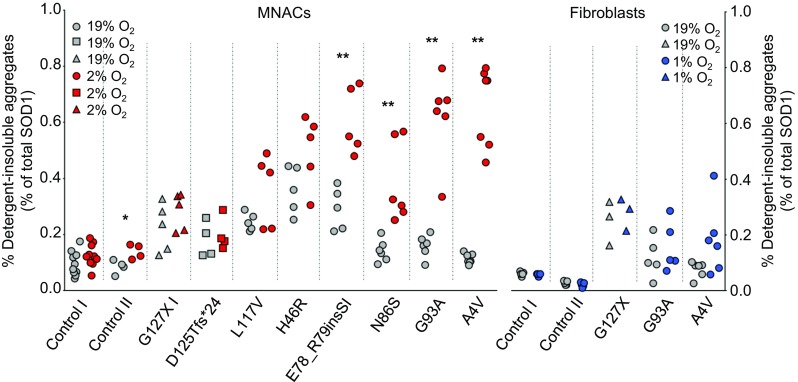
Low O_2_ tension promotes SOD1 aggregation in MNACs but not in fibroblasts. Scatter plots show the amounts of SOD1 present in the detergent-insoluble fraction determined by western blotting as a % of soluble SOD1 present in the cell extract determined by total SOD1 ELISA: MNACs (red; 2% O_2_ for 24 h) and fibroblasts (blue; 1% O_2_ for 24 h) in comparison to replicate cultures (grey; 19% O_2_ for 24 h). Full-length SOD1s are depicted as filled circles and quantified using an anti-SOD1 (aa 57–72) antibody (Supplementary Figs. 10a and 11a). The SOD1^G127X^ mutant protein (filled triangles) was quantified using a hSOD1^G127X^-specific antibody (Supplementary Figs. 10b and 11b). The SOD1^D125Tfs*24^ mutant protein (filled squares) was quantified using the aa 57–72 antibody (Supplementary Figs. 10a and 11a). The identity of the mutant protein band was confirmed by lack of reactivity against a C-terminal SOD1 (aa 143–153) peptide (Supplementary Fig. 11c). Data are expressed as the mean ± SD of four to ten technical replicates from two to six independent MNACs differentiations, and of five to six technical replicates from two to three independent experiments for fibroblasts, with the exception of the SOD1^G127X^ fibroblast line where data are expressed as the mean ± SD of three technical replicates from one experiment;*p < 0.05, **p < 0.01, analyzed by Mann–Whitney U test

Low levels of aggregation were found in fibroblasts, which did not increase significantly in response to low O_2_ (Fig. 8). In contrast, we found that low O_2_ tension induced marked increases in aggregation in MNACs carrying full-length mutant SOD1s (E78_R79insSI, N86S, G93A and A4V), but not in SOD1^WT^ control lines or SOD1^L117V^, which has SOD1^WT^-like stability [71]. The *SOD1*^*H46R*^ mutation disrupts copper binding catalyzed via CCS and impairs formation of the disulfide bond [75]. In agreement, the high level of aggregation present at 19% O_2_ in the SOD1^H46R^ line did not change at 2% O_2_. Neither of the lines expressing the C-terminally truncated mutants that lack the disulfide bond (SOD1^G127X^ and SOD1^D125Tfs*24^), showed increased aggregation. Hence, increased aggregation correlated closely with increased disorder in MNACs expressing full-length mutant SOD1s.

## Discussion

By comparing the responses of a large panel of patient-derived cell lines carrying genetically and biochemically distinct SOD1 variants, we have found that low O_2_ tension leads to remarkably large increases in SOD1 C57–C146 disulfide bond reduction and disorder. In addition to being required for bond formation, we have identified that O_2_ is also critical for its maintenance. Furthermore, low O_2_ tension resulted in enhanced mutant SOD1 aggregation in MNACs but not in fibroblasts. This supports the idea that in human patient-derived models, as well as in Tg mice, the C57–C146 disulfide bond is an ALS-related Achilles heel of SOD1 in the reducing environment of the cytosol.

The O_2_ tensions found to enhance disordered SOD1 are within the range considered to be normoxic in human and animal tissues. However, the range of O_2_ tensions present within cultured cells is likely to be lower. This is due to both the rate of O_2_ diffusion through the culture medium and the rate of O_2_ consumption by cellular respiration, which depends on both cellular mass and the rate of oxidative metabolism [61]. Because these variables are difficult to control for between different cell lines, we chose to compare replicate samples cultured at different O_2_ tensions. The cells, which had been propagated and adapted to growth at 19% O_2_, were tested within a range that was found not to cause overt toxicity. A study in young resting awake mice found that O_2_ tensions in the CNS vary between 0.2 and 5% [55]. Even lower levels were recorded under normal conditions, e.g. in periarteriolar areas. Considering this great variation found in young mice, it is reasonable to assume that localized hypoxia could occur transiently, or more chronically, in middle-aged or elderly humans even in the absence of overt disease.

In *SOD1* ALS mouse models, the average lifespan of homozygous Tg mice is approximately half that of hemizygous animals [44, 45]. In human ALS there is also a dose-dependence on mutant SOD1; doubling of the load of mutant SOD1 in individuals homozygous for *SOD1* mutations L84F, N86S or L126S results in earlier onset and more rapid progression than in the heterozygous state [1, 16, 42, 49]. Furthermore, the D90A mutant protein, which has wild-type-like stability, typically only causes ALS in homozygous individuals [5]. Therefore, if our results showing large increases in disordered SOD1 in MNACs in response to low O_2_ tensions translate to the human CNS, this could likely contribute to the initiation and progression of ALS.

Recent findings in vivo suggest that prion-like growth and spread of SOD1 aggregation could be the primary disease mechanism of SOD1-induced ALS [7, 8, 12, 28]. Disordered SOD1 species are critical substrates for both the nucleation and growth of aggregates [21, 22, 35, 51, 53]. Typically, in animal or cell models of ALS, SOD1 overexpression is required for substantial aggregation to occur [63]. Our results show that in patient-derived MNACs that do not overexpress SOD1, even a short (24 h) exposure to low O_2_ tension can induce large increases in mutant SOD1 aggregation. This suggests that in patients SOD1 aggregation would have a greater probability of initiation in areas of the CNS with sustained low O_2_ tension.

As to the impact of low O_2_ tension on animal models, van den Bosch et al. [73] found no effect on the lifespan of hSOD1^G93A^ Gur Tg mice exposed to 10% O_2_ at early or late symptomatic stages [73]. However, even when housed in normoxic conditions (21% O_2_), a significant proportion of mutant SOD1 in the spinal cord of hSOD1^G93A^ mice is disulfide-reduced due to the extreme level of overexpression in this line [46]. Moreover, exponential aggregation of hSOD1^G93A^ begins even before birth and has advanced considerably by the stages that mice were exposed to low O_2_ in the van den Bosch study [11]. Hence, it is unlikely that the effects that we have found in human patient-derived models in this study would be detectable in the hSOD1^G93A^ Tg model.

Significant increases in disulfide-reduced and disordered SOD1 were also seen in cells from healthy control individuals and cells derived from patients carrying ALS-linked mutations in other genes. Although we only detected a significant increase in the aggregation of mutant and not SOD1^WT^ over this short period, this could be important given the mounting evidence for the involvement of SOD1^WT^ in ALS.

In summary, we show that O_2_ tension is a principal determinant of SOD1 disorder and aggregation. Our findings suggest that CNS areas with low O_2_ tension could act as foci for the initiation or progression of ALS. This mechanism might contribute to the enhanced risk for the disease associated with aging, as well as other factors that impair vascular perfusion.

## Electronic supplementary material

Below is the link to the electronic supplementary material.

Supplementary material 1 (PPTX 8160 kb)
