# Supplementary material for: The molecular pathogenesis of superoxide dismutase 1-linked ALS is promoted by low oxygen tension
Source: Acta Neuropathol. 2019 Mar 12;138(1):85–101. doi: 10.1007/s00401-019-01986-1 (PMC6570705; doi:10.1007/s00401-019-01986-1)
Supplement: Supplementary file 1 — Supplementary material 1 (PPTX 8160 kb) [file 401_2019_1986_MOESM1_ESM.pptx]

## Slide 1
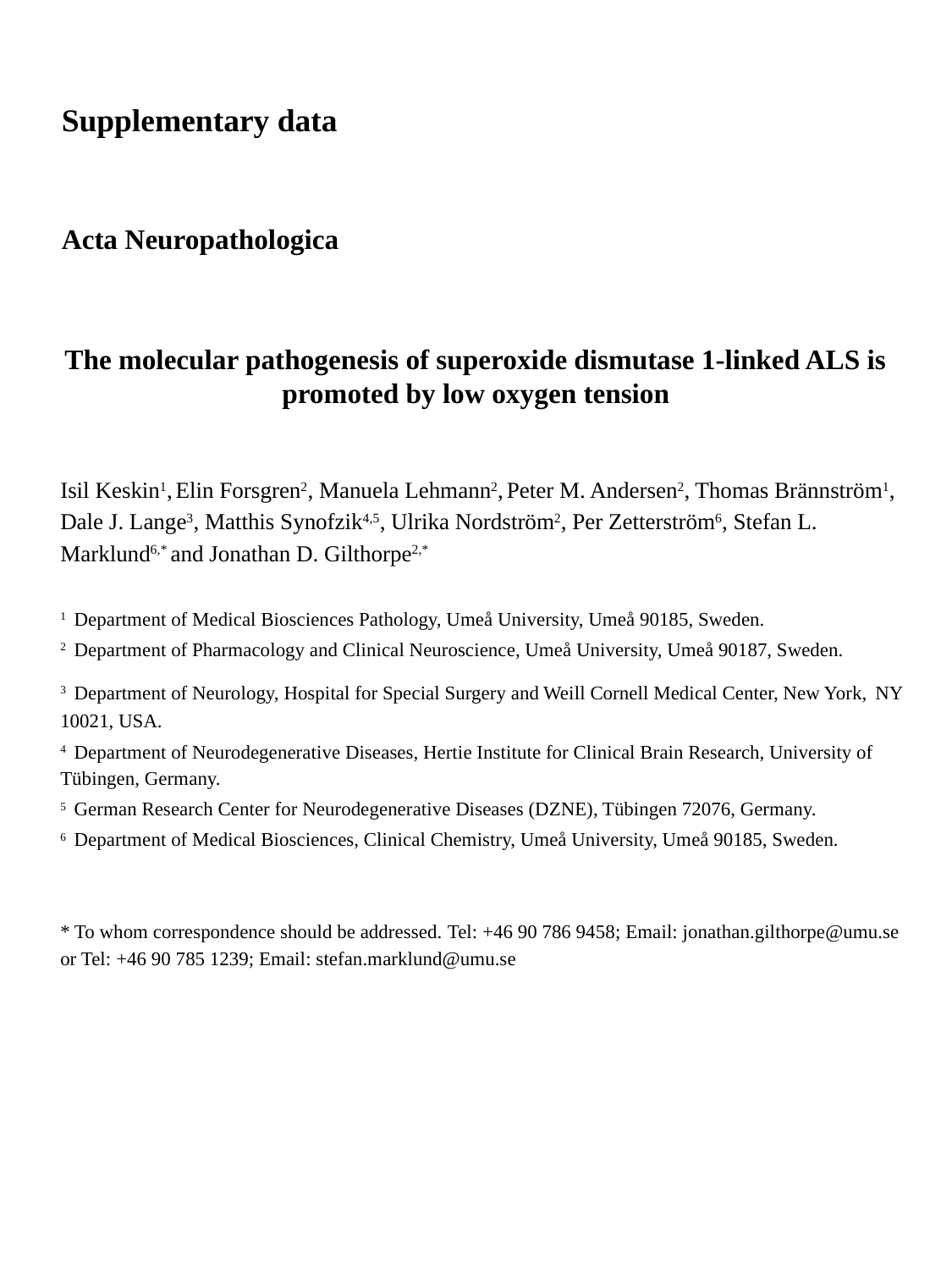

Supplementary data
Acta Neuropathologica
# The molecular pathogenesis of superoxide dismutase 1-linked ALS is promoted by low oxygen tension
Isil Keskin1, Elin Forsgren2, Manuela Lehmann2, Peter M. Andersen2, Thomas Brännström1, Dale J. Lange3, Matthis Synofzik4,5, Ulrika Nordström2, Per Zetterström6, Stefan L. Marklund6,* and Jonathan D. Gilthorpe2,*
1 Department of Medical Biosciences Pathology, Umeå University, Umeå 90185, Sweden.
2 Department of Pharmacology and Clinical Neuroscience, Umeå University, Umeå 90187, Sweden.
3 Department of Neurology, Hospital for Special Surgery and Weill Cornell Medical Center, New York, NY 10021, USA.
4 Department of Neurodegenerative Diseases, Hertie Institute for Clinical Brain Research, University of Tübingen, Germany.
5 German Research Center for Neurodegenerative Diseases (DZNE), Tübingen 72076, Germany.
6 Department of Medical Biosciences, Clinical Chemistry, Umeå University, Umeå 90185, Sweden.
* To whom correspondence should be addressed. Tel: +46 90 786 9458; Email: jonathan.gilthorpe@umu.se or Tel: +46 90 785 1239; Email: stefan.marklund@umu.se

## Slide 2
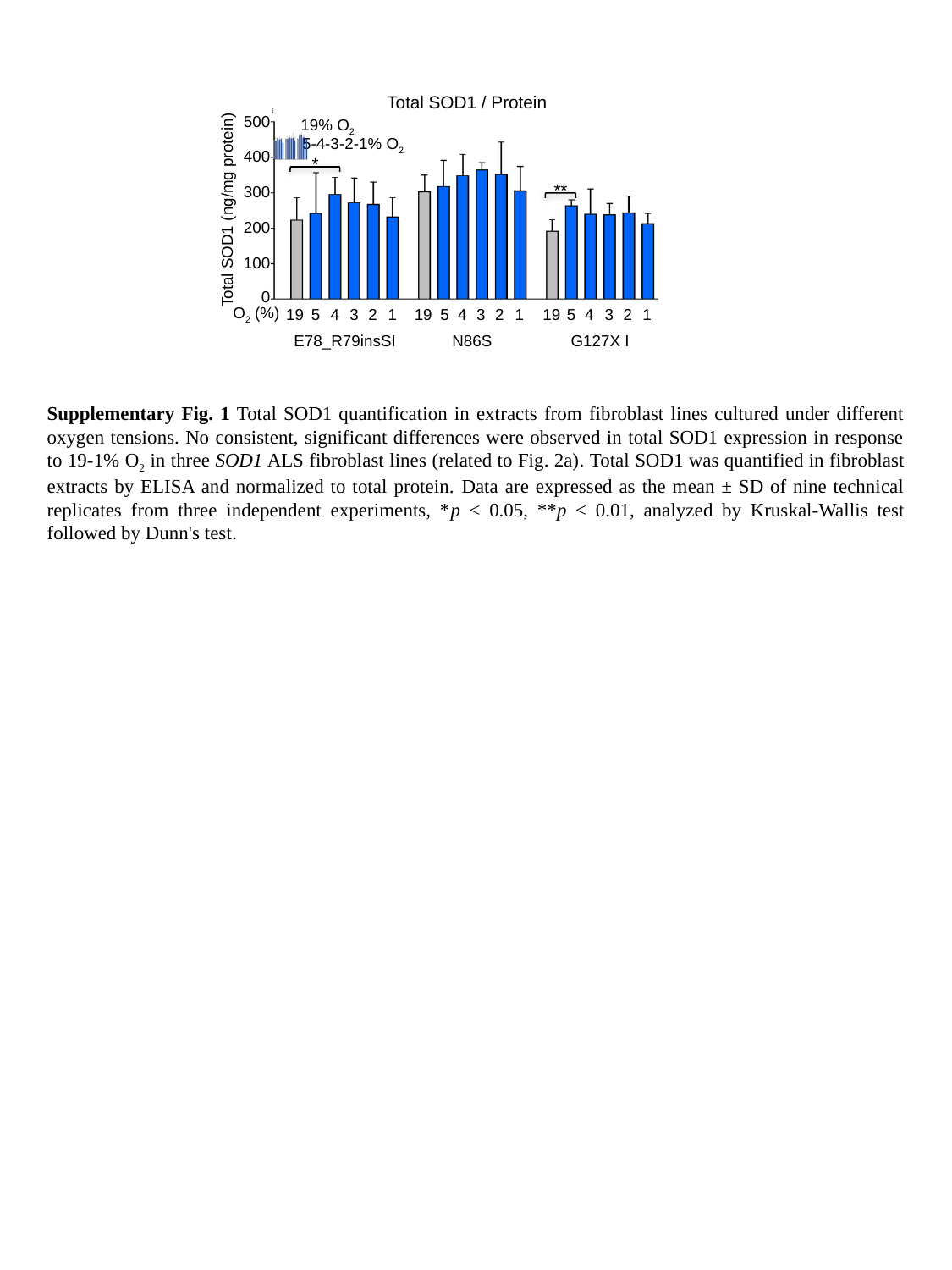

Total SOD1 / Protein
500
400
300
200
100
0
19% O2
5-4-3-2-1% O2
*
**
Total SOD1 (ng/mg protein)
O2 (%)
19
5
4
3
2
1
19
5
4
3
2
1
19
5
4
3
2
1
E78_R79insSI
N86S
G127X I
Supplementary Fig. 1 Total SOD1 quantification in extracts from fibroblast lines cultured under different oxygen tensions. No consistent, significant differences were observed in total SOD1 expression in response to 19-1% O2 in three SOD1 ALS fibroblast lines (related to Fig. 2a). Total SOD1 was quantified in fibroblast extracts by ELISA and normalized to total protein. Data are expressed as the mean ± SD of nine technical replicates from three independent experiments, *p < 0.05, **p < 0.01, analyzed by Kruskal-Wallis test followed by Dunn's test.

## Slide 3
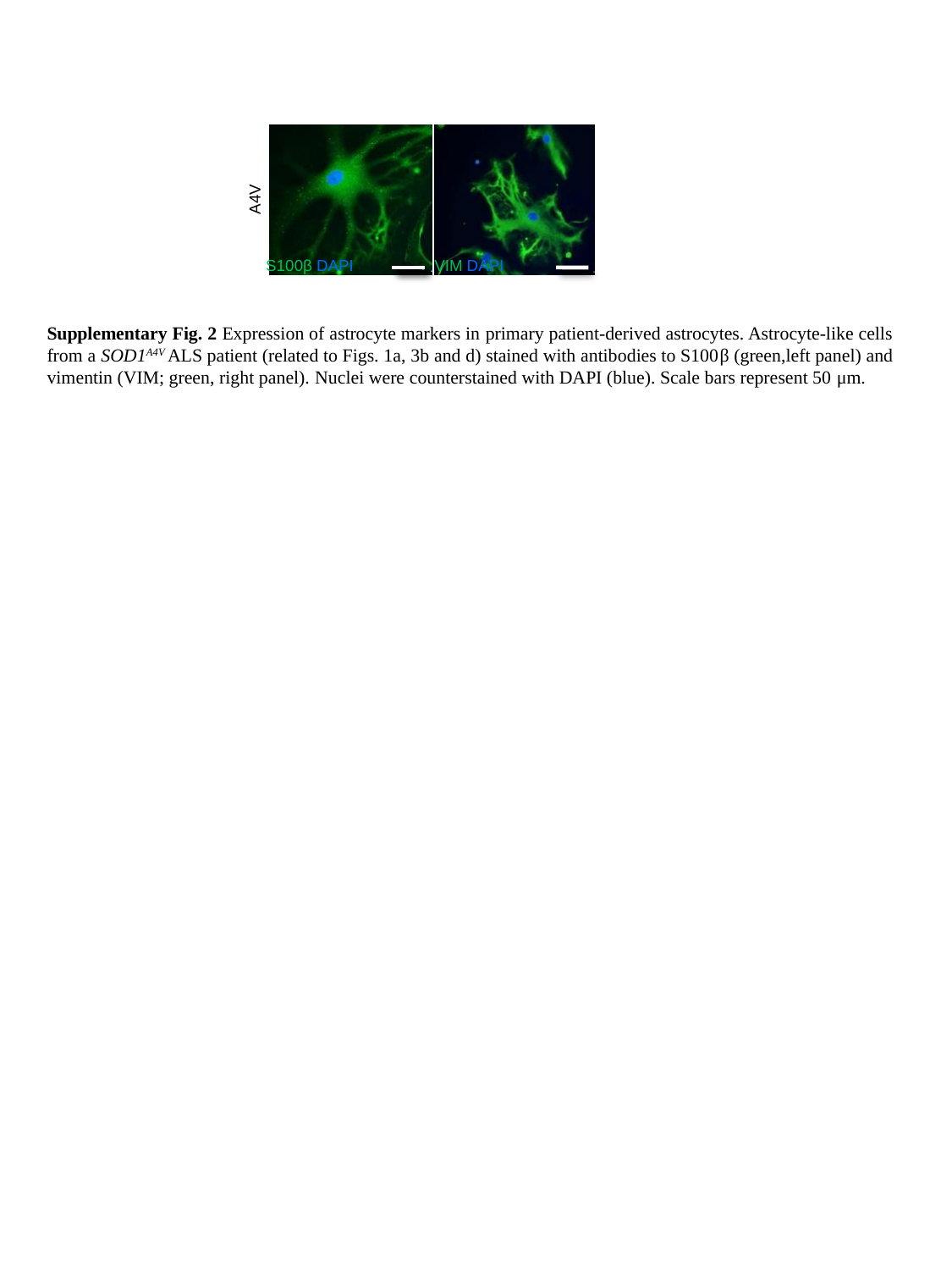

A4V
S100β DAPI
VIM DAPI
Supplementary Fig. 2 Expression of astrocyte markers in primary patient-derived astrocytes. Astrocyte-like cells from a SOD1A4V ALS patient (related to Figs. 1a, 3b and d) stained with antibodies to S100β (green,left panel) and vimentin (VIM; green, right panel). Nuclei were counterstained with DAPI (blue). Scale bars represent 50 μm.

## Slide 4
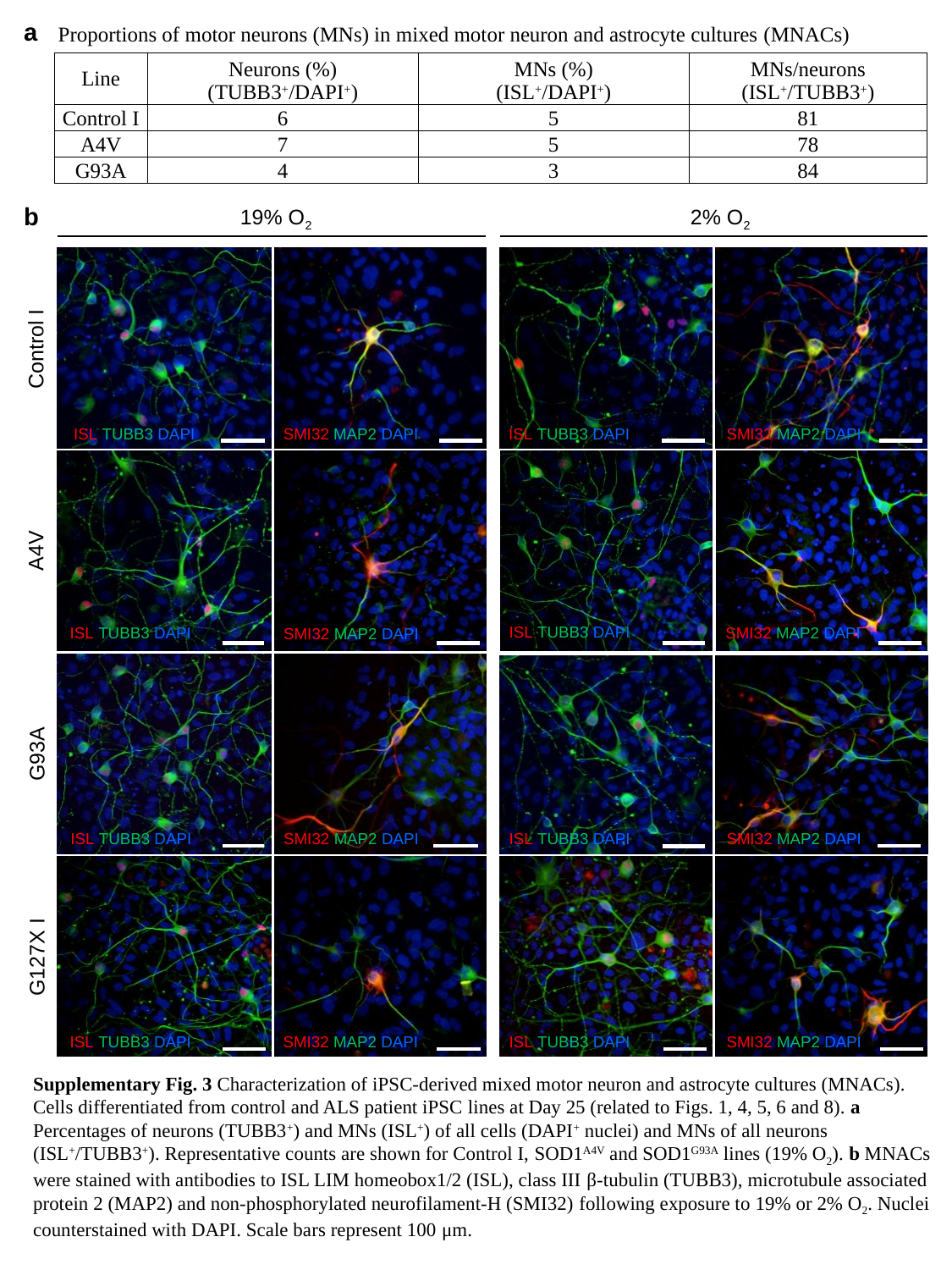

a
Proportions of motor neurons (MNs) in mixed motor neuron and astrocyte cultures (MNACs)
| Line | Neurons (%) (TUBB3+/DAPI+) | MNs (%) (ISL+/DAPI+) | MNs/neurons (ISL+/TUBB3+) |
| --- | --- | --- | --- |
| Control I | 6 | 5 | 81 |
| A4V | 7 | 5 | 78 |
| G93A | 4 | 3 | 84 |
b
19% O2
2% O2
ISL TUBB3 DAPI
SMI32 MAP2 DAPI
ISL TUBB3 DAPI
SMI32 MAP2 DAPI
Control I
ISL TUBB3 DAPI
SMI32 MAP2 DAPI
ISL TUBB3 DAPI
SMI32 MAP2 DAPI
A4V
ISL TUBB3 DAPI
SMI32 MAP2 DAPI
ISL TUBB3 DAPI
SMI32 MAP2 DAPI
G93A
ISL TUBB3 DAPI
SMI32 MAP2 DAPI
ISL TUBB3 DAPI
SMI32 MAP2 DAPI
G127X I
Supplementary Fig. 3 Characterization of iPSC-derived mixed motor neuron and astrocyte cultures (MNACs). Cells differentiated from control and ALS patient iPSC lines at Day 25 (related to Figs. 1, 4, 5, 6 and 8). a Percentages of neurons (TUBB3+) and MNs (ISL+) of all cells (DAPI+ nuclei) and MNs of all neurons (ISL+/TUBB3+). Representative counts are shown for Control I, SOD1A4V and SOD1G93A lines (19% O2). b MNACs were stained with antibodies to ISL LIM homeobox1/2 (ISL), class III β-tubulin (TUBB3), microtubule associated protein 2 (MAP2) and non-phosphorylated neurofilament-H (SMI32) following exposure to 19% or 2% O2. Nuclei counterstained with DAPI. Scale bars represent 100 μm.

## Slide 5
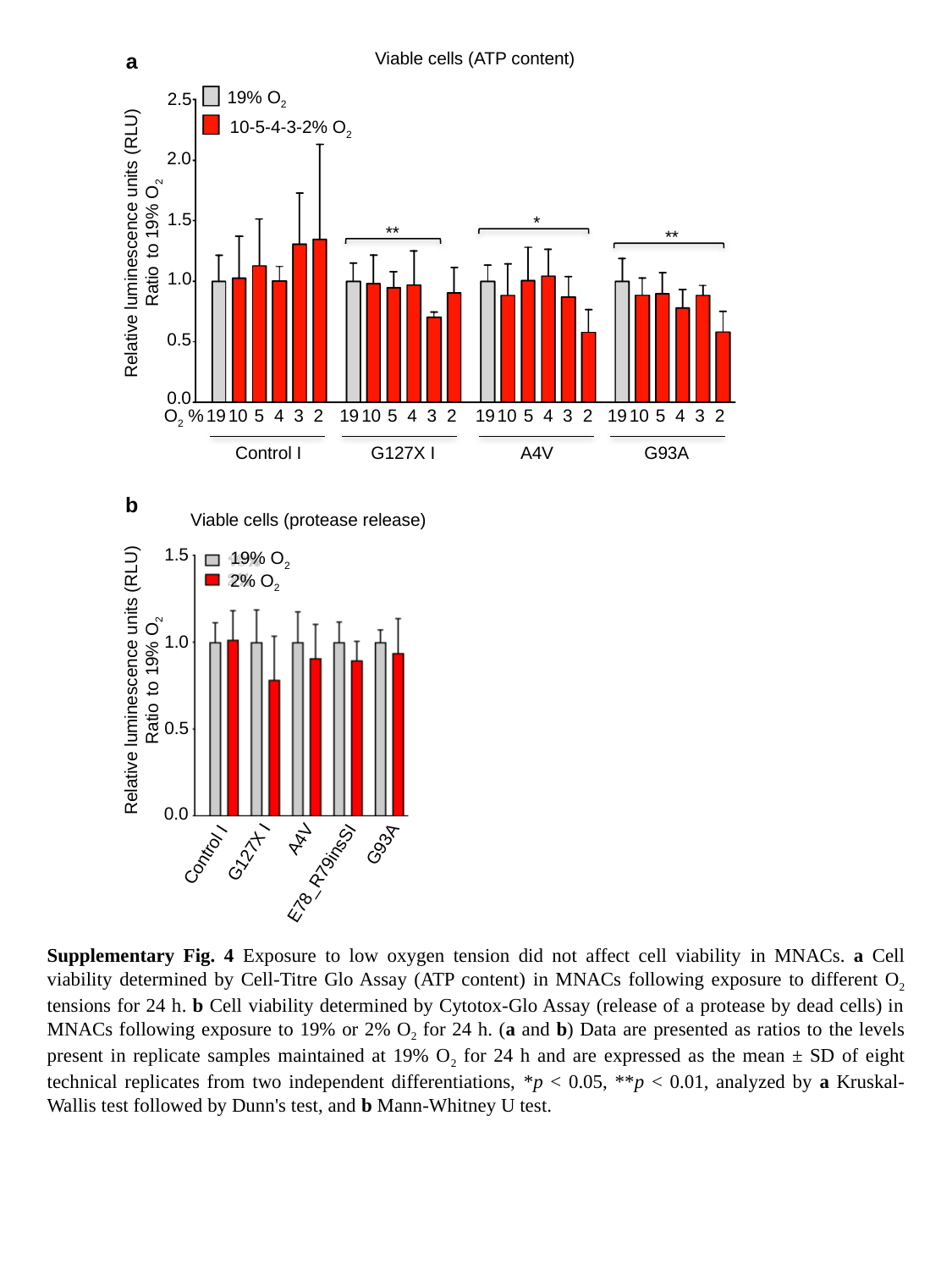

Viable cells (ATP content)
a
19% O2
2.5
2.0
1.5
1.0
0.5
0.0
10-5-4-3-2% O2
*
**
Relative luminescence units (RLU)
Ratio to 19% O2
**
O2 %
19
10
5
4
3
2
19
10
5
4
3
2
19
10
5
4
3
2
19
10
5
4
3
2
Control I
G127X I
A4V
G93A
b
Viable cells (protease release)
1.5
1.0
0.5
0.0
19% O2
2% O2
Relative luminescence units (RLU)
Ratio to 19% O2
A4V
G93A
G127X I
Control I
E78_R79insSI
Supplementary Fig. 4 Exposure to low oxygen tension did not affect cell viability in MNACs. a Cell viability determined by Cell-Titre Glo Assay (ATP content) in MNACs following exposure to different O2 tensions for 24 h. b Cell viability determined by Cytotox-Glo Assay (release of a protease by dead cells) in MNACs following exposure to 19% or 2% O2 for 24 h. (a and b) Data are presented as ratios to the levels present in replicate samples maintained at 19% O2 for 24 h and are expressed as the mean ± SD of eight technical replicates from two independent differentiations, *p < 0.05, **p < 0.01, analyzed by a Kruskal-Wallis test followed by Dunn's test, and b Mann-Whitney U test.

## Slide 6
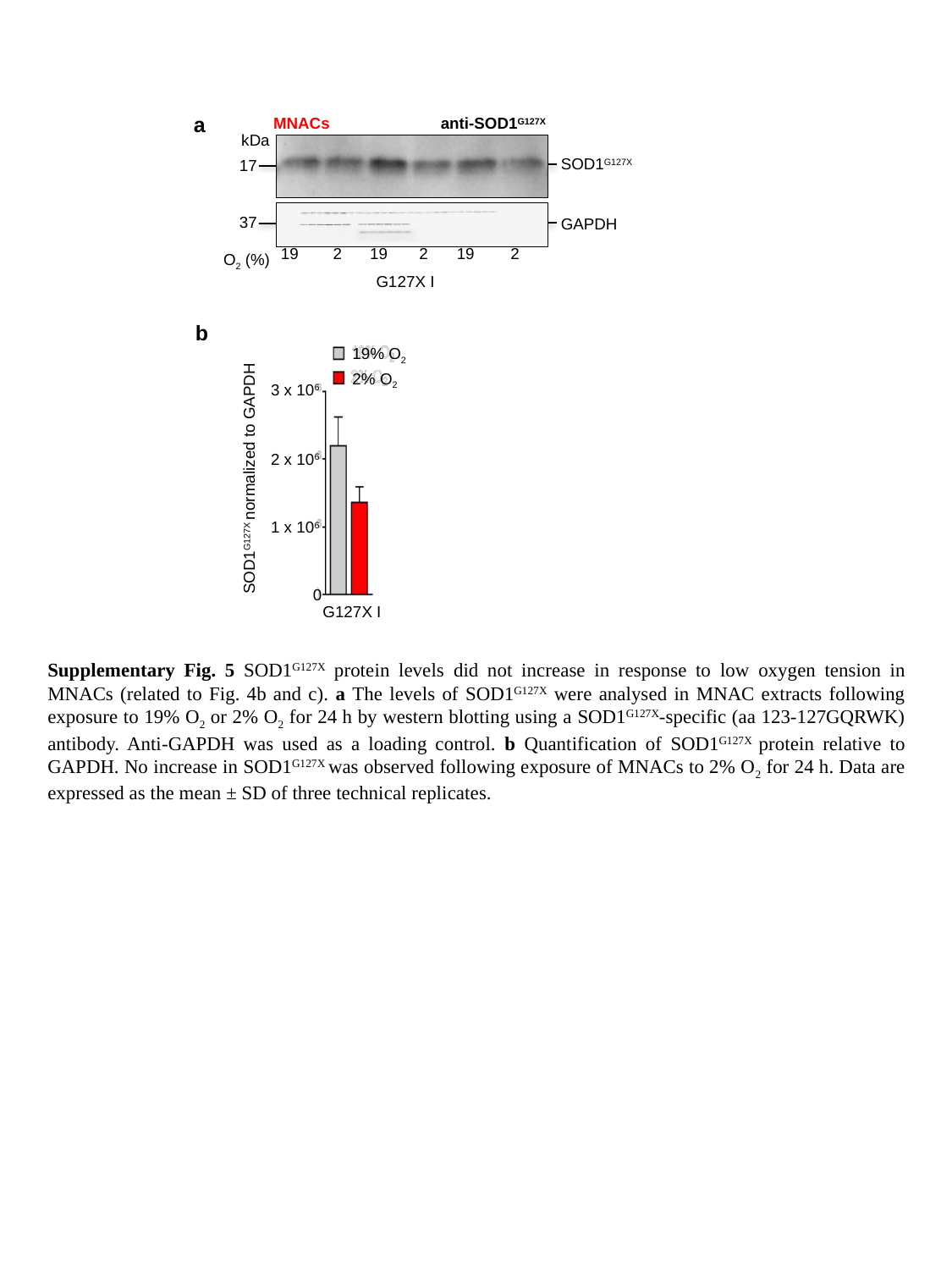

a
MNACs
anti-SOD1G127X
kDa
SOD1G127X
17
37
GAPDH
G127X I
19
19
19
2
2
2
O2 (%)
b
19% O2
2% O2
3 x 10^6^
2 x 10^6^
SOD1G127X normalized to GAPDH
1 x 10^6^
G127X I
0
Supplementary Fig. 5 SOD1G127X protein levels did not increase in response to low oxygen tension in MNACs (related to Fig. 4b and c). a The levels of SOD1G127X were analysed in MNAC extracts following exposure to 19% O2 or 2% O2 for 24 h by western blotting using a SOD1G127X-specific (aa 123-127GQRWK) antibody. Anti-GAPDH was used as a loading control. b Quantification of SOD1G127X protein relative to GAPDH. No increase in SOD1G127X was observed following exposure of MNACs to 2% O2 for 24 h. Data are expressed as the mean ± SD of three technical replicates.

## Slide 7
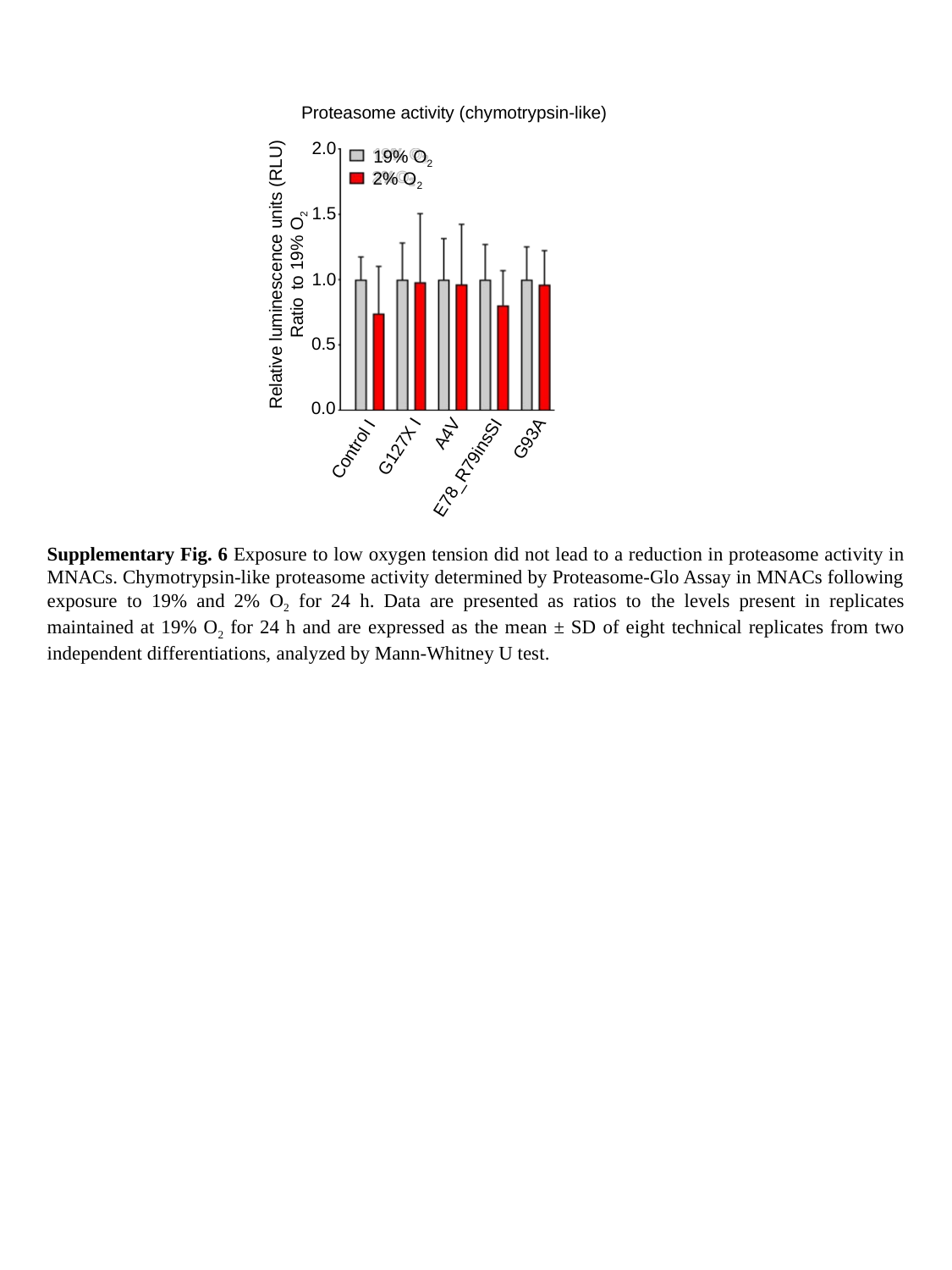

Proteasome activity (chymotrypsin-like)
2.0
19% O2
2% O2
1.5
Relative luminescence units (RLU)
Ratio to 19% O2
1.0
0.5
0.0
A4V
G93A
G127X I
Control I
E78_R79insSI
Supplementary Fig. 6 Exposure to low oxygen tension did not lead to a reduction in proteasome activity in MNACs. Chymotrypsin-like proteasome activity determined by Proteasome-Glo Assay in MNACs following exposure to 19% and 2% O2 for 24 h. Data are presented as ratios to the levels present in replicates maintained at 19% O2 for 24 h and are expressed as the mean ± SD of eight technical replicates from two independent differentiations, analyzed by Mann-Whitney U test.

## Slide 8
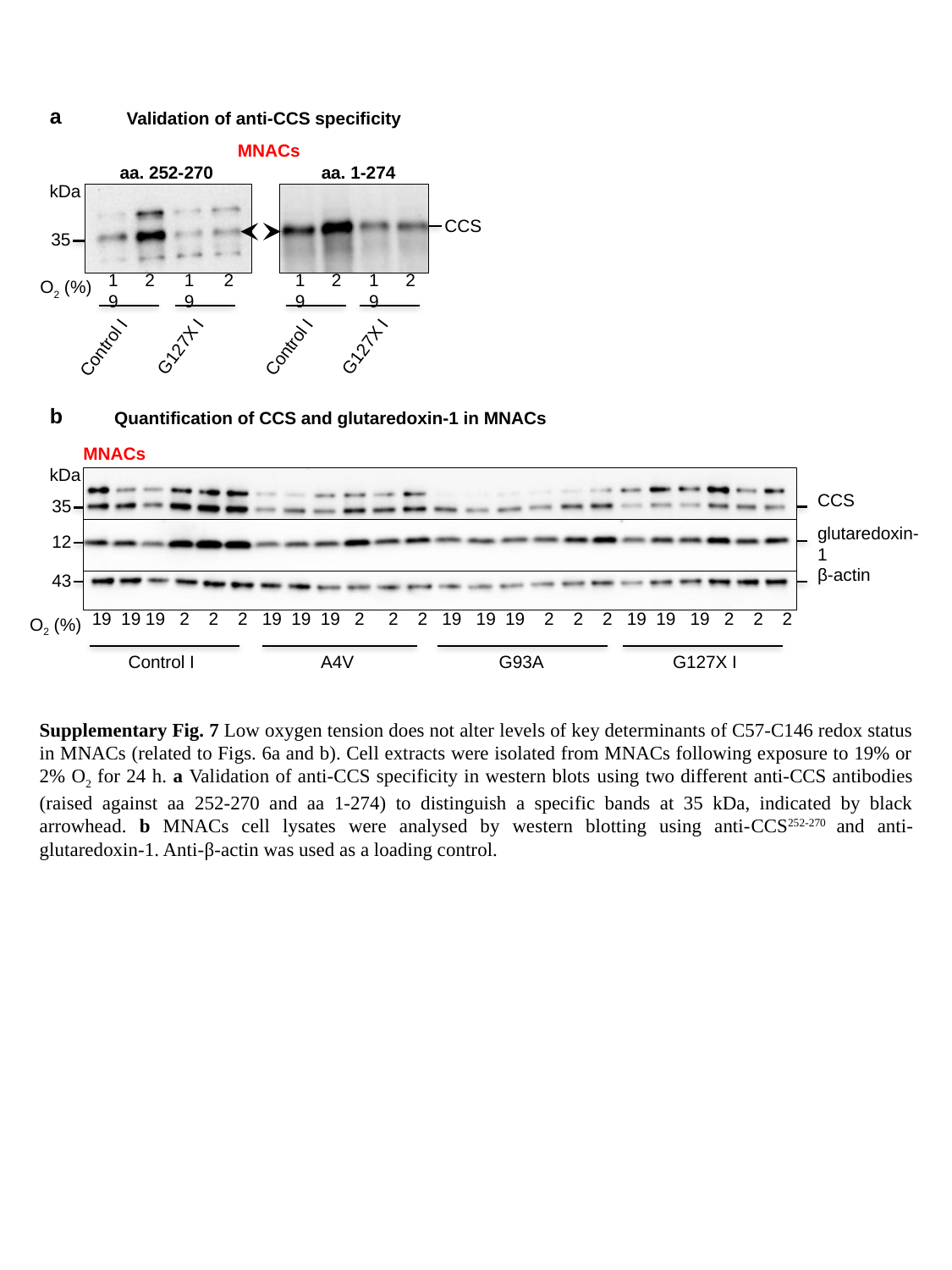

a
Validation of anti-CCS specificity
MNACs
aa. 252-270
aa. 1-274
kDa
CCS
35
19 19 19 2 2 2 19 19 19 2 2 2 19 19 19 2 2 2 19 19 19 2 2 2
2
2
2
2
19
19
19
19
O2 (%)
Control I
G127X I
G127X I
Control I
b
Quantification of CCS and glutaredoxin-1 in MNACs
MNACs
kDa
CCS
glutaredoxin-1
35
β-actin
12
43
Control I
G127X I
A4V
G93A
O2 (%)
Supplementary Fig. 7 Low oxygen tension does not alter levels of key determinants of C57-C146 redox status in MNACs (related to Figs. 6a and b). Cell extracts were isolated from MNACs following exposure to 19% or 2% O2 for 24 h. a Validation of anti-CCS specificity in western blots using two different anti-CCS antibodies (raised against aa 252-270 and aa 1-274) to distinguish a specific bands at 35 kDa, indicated by black arrowhead. b MNACs cell lysates were analysed by western blotting using anti-CCS252-270 and anti-glutaredoxin-1. Anti-β-actin was used as a loading control.

## Slide 9
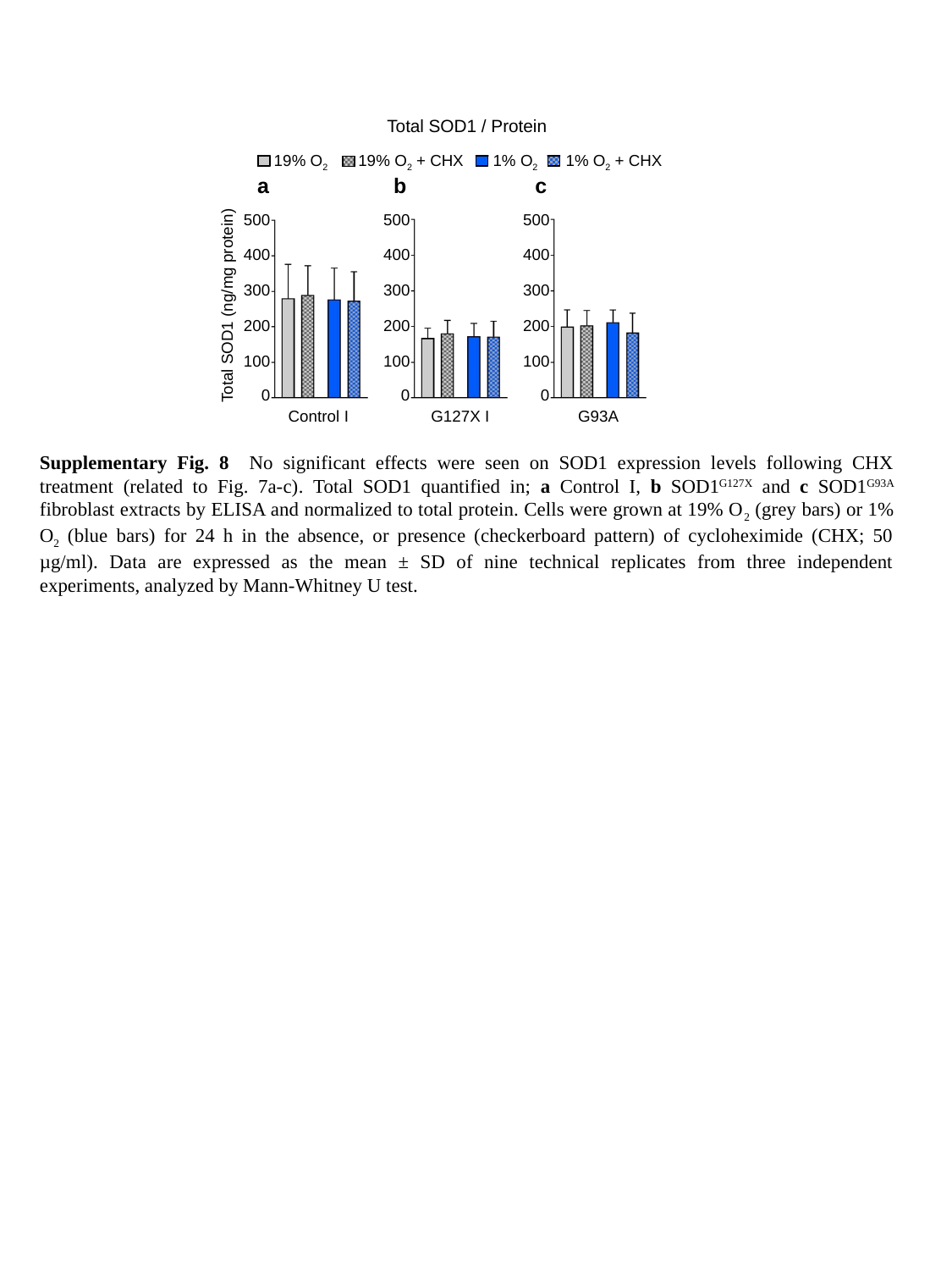

Total SOD1 / Protein
19% O2
19% O2 + CHX
1% O2
1% O2 + CHX
a
b
c
500
400
300
200
100
0
500
400
300
200
100
0
500
400
300
200
100
0
Total SOD1 (ng/mg protein)
Control I
G127X I
G93A
Supplementary Fig. 8 No significant effects were seen on SOD1 expression levels following CHX treatment (related to Fig. 7a-c). Total SOD1 quantified in; a Control I, b SOD1G127X and c SOD1G93A fibroblast extracts by ELISA and normalized to total protein. Cells were grown at 19% O2 (grey bars) or 1% O2 (blue bars) for 24 h in the absence, or presence (checkerboard pattern) of cycloheximide (CHX; 50 µg/ml). Data are expressed as the mean ± SD of nine technical replicates from three independent experiments, analyzed by Mann-Whitney U test.

## Slide 10
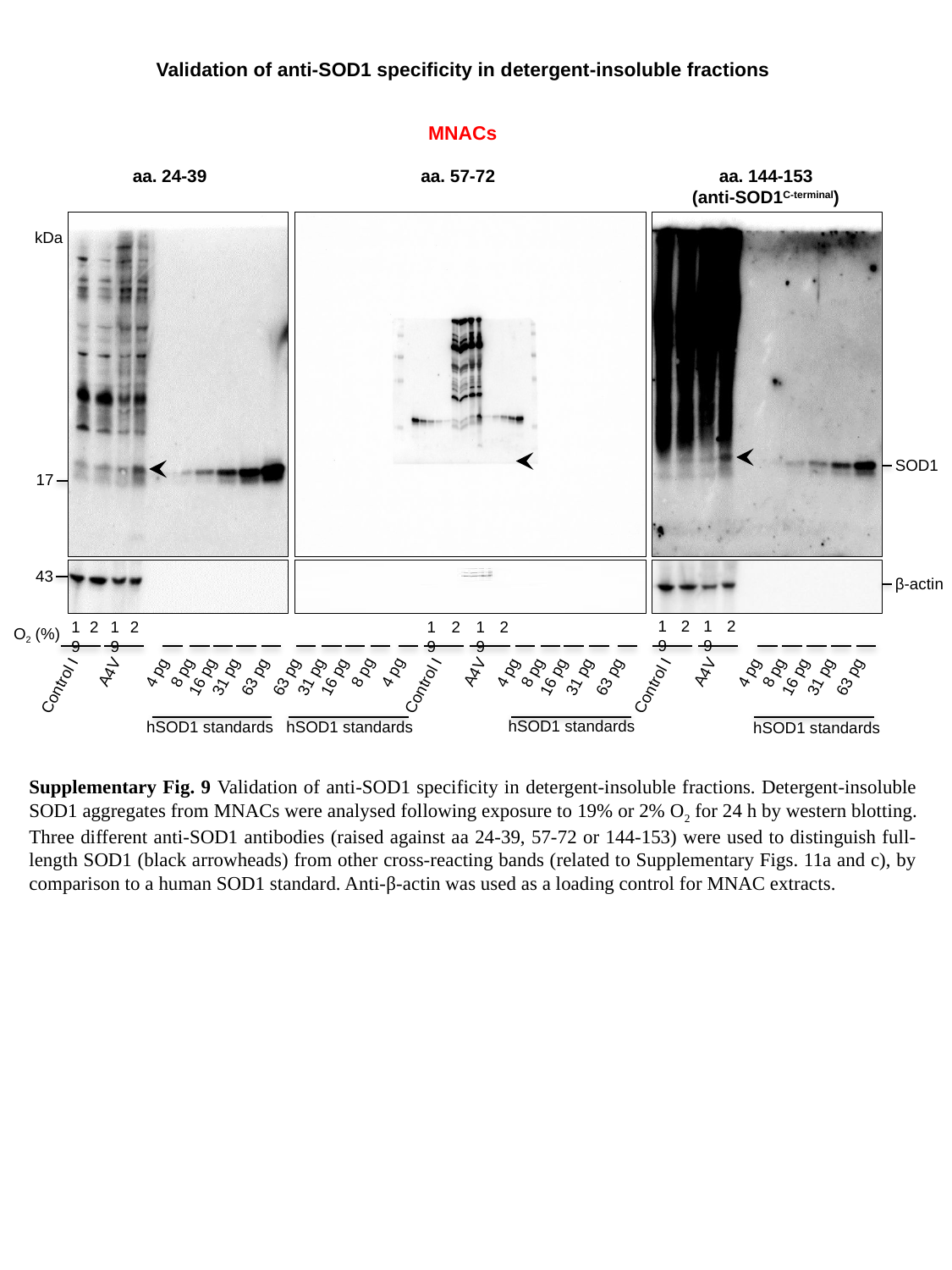

Validation of anti-SOD1 specificity in detergent-insoluble fractions
MNACs
aa. 24-39
aa. 57-72
aa. 144-153
(anti-SOD1C-terminal)
kDa
SOD1
17
43
β-actin
2
2
2
2
2
2
19
19
19
19
19
19
O2 (%)
hSOD1 standards
hSOD1 standards
hSOD1 standards
hSOD1 standards
Control I
A4V
4 pg
63 pg
Control I
A4V
4 pg
Control I
A4V
4 pg
31 pg
8 pg
31 pg
63 pg
4 pg
63 pg
63 pg
16 pg
16 pg
16 pg
16 pg
8 pg
31 pg
8 pg
8 pg
31 pg
Supplementary Fig. 9 Validation of anti-SOD1 specificity in detergent-insoluble fractions. Detergent-insoluble SOD1 aggregates from MNACs were analysed following exposure to 19% or 2% O2 for 24 h by western blotting. Three different anti-SOD1 antibodies (raised against aa 24-39, 57-72 or 144-153) were used to distinguish full-length SOD1 (black arrowheads) from other cross-reacting bands (related to Supplementary Figs. 11a and c), by comparison to a human SOD1 standard. Anti-β-actin was used as a loading control for MNAC extracts.

## Slide 11
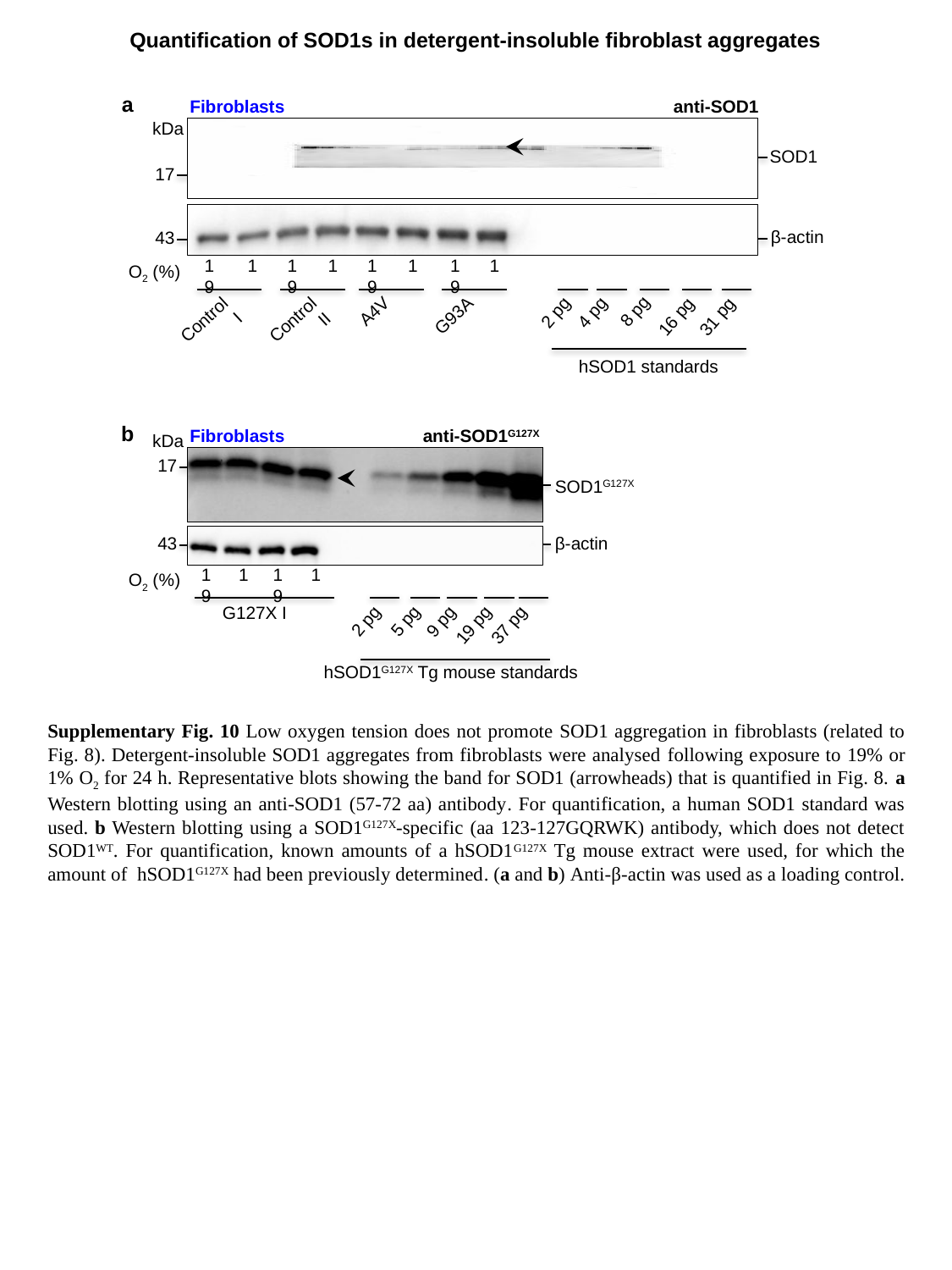

Quantification of SOD1s in detergent-insoluble fibroblast aggregates
a
Fibroblasts
anti-SOD1
kDa
SOD1
17
β-actin
43
1
1
1
1
19
19
19
19
O2 (%)
8 pg
4 pg
2 pg
31 pg
16 pg
G93A
Control II
Control I
A4V
hSOD1 standards
b
Fibroblasts
anti-SOD1G127X
kDa
17
SOD1G127X
hSOD1G127X Tg mouse standards
β-actin
43
G127X I
1
1
19
19
O2 (%)
5 pg
2 pg
19 pg
37 pg
9 pg
Supplementary Fig. 10 Low oxygen tension does not promote SOD1 aggregation in fibroblasts (related to Fig. 8). Detergent-insoluble SOD1 aggregates from fibroblasts were analysed following exposure to 19% or 1% O2 for 24 h. Representative blots showing the band for SOD1 (arrowheads) that is quantified in Fig. 8. a Western blotting using an anti-SOD1 (57-72 aa) antibody. For quantification, a human SOD1 standard was used. b Western blotting using a SOD1G127X-specific (aa 123-127GQRWK) antibody, which does not detect SOD1WT. For quantification, known amounts of a hSOD1G127X Tg mouse extract were used, for which the amount of hSOD1G127X had been previously determined. (a and b) Anti-β-actin was used as a loading control.

## Slide 12
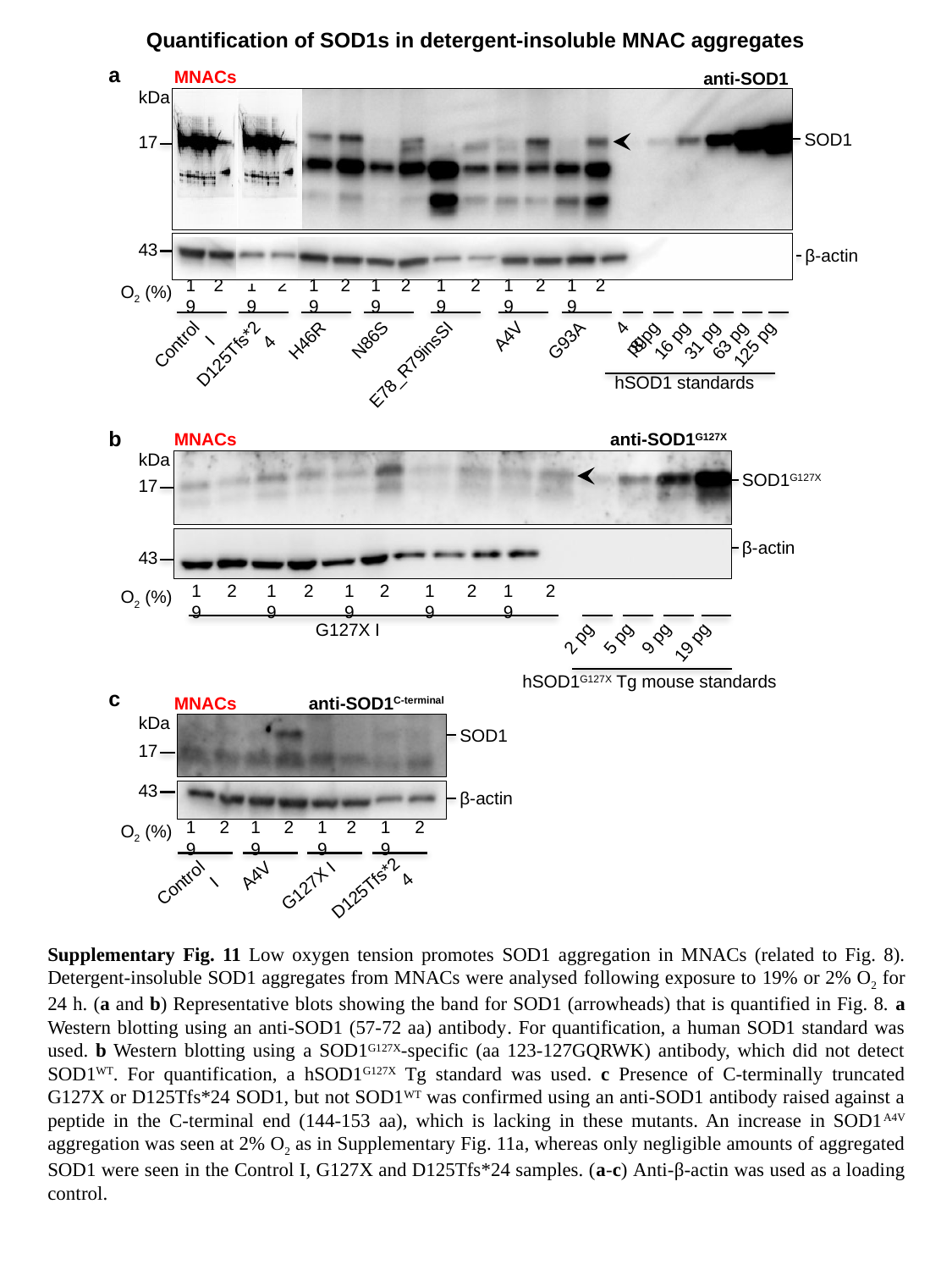

Quantification of SOD1s in detergent-insoluble MNAC aggregates
a
MNACs
anti-SOD1
kDa
SOD1
17
43
β-actin
2
2
2
2
2
2
2
19
19
19
19
19
19
19
O2 (%)
hSOD1 standards
E78_R79insSI
A4V
D125Tfs*24
16 pg
63 pg
125 pg
31 pg
G93A
Control I
N86S
H46R
8 pg
4 pg
b
MNACs
anti-SOD1G127X
kDa
SOD1G127X
17
β-actin
hSOD1G127X Tg mouse standards
43
2
2
2
2
2
G127X I
19
19
19
19
19
O2 (%)
5 pg
2 pg
19 pg
9 pg
c
MNACs
anti-SOD1C-terminal
kDa
SOD1
17
43
β-actin
2
2
2
2
O2 (%)
19
19
19
19
D125Tfs*24
A4V
Control I
G127X I
Supplementary Fig. 11 Low oxygen tension promotes SOD1 aggregation in MNACs (related to Fig. 8). Detergent-insoluble SOD1 aggregates from MNACs were analysed following exposure to 19% or 2% O2 for 24 h. (a and b) Representative blots showing the band for SOD1 (arrowheads) that is quantified in Fig. 8. a Western blotting using an anti-SOD1 (57-72 aa) antibody. For quantification, a human SOD1 standard was used. b Western blotting using a SOD1G127X-specific (aa 123-127GQRWK) antibody, which did not detect SOD1WT. For quantification, a hSOD1G127X Tg standard was used. c Presence of C-terminally truncated G127X or D125Tfs*24 SOD1, but not SOD1WT was confirmed using an anti-SOD1 antibody raised against a peptide in the C-terminal end (144-153 aa), which is lacking in these mutants. An increase in SOD1A4V aggregation was seen at 2% O2 as in Supplementary Fig. 11a, whereas only negligible amounts of aggregated SOD1 were seen in the Control I, G127X and D125Tfs*24 samples. (a-c) Anti-β-actin was used as a loading control.

## Slide 13
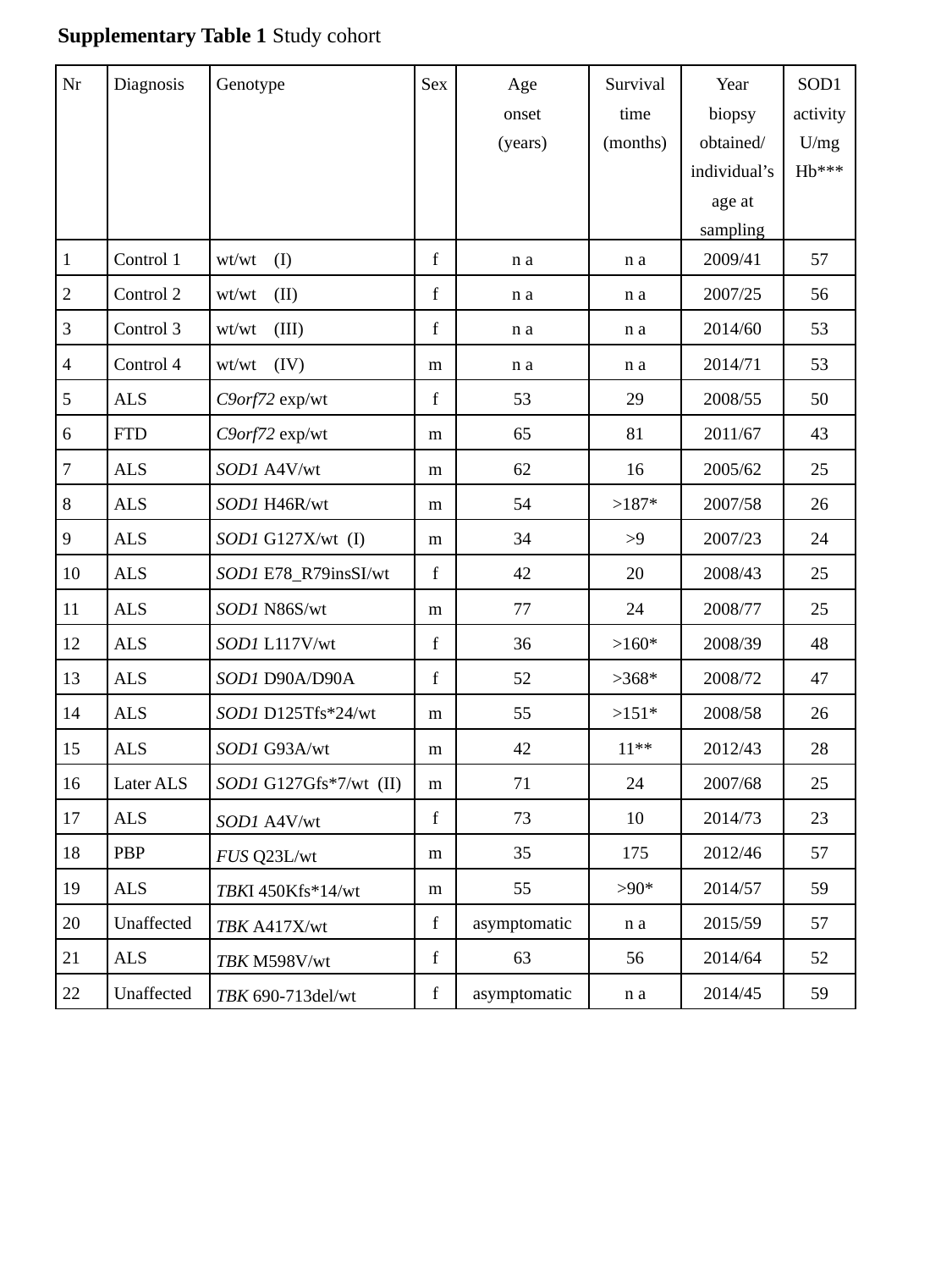

Supplementary Table 1 Study cohort
| Nr | Diagnosis | Genotype | Sex | Age onset (years) | Survival time (months) | Year biopsy obtained/ individual’s age at sampling | SOD1 activity U/mg Hb\*\*\* |
| --- | --- | --- | --- | --- | --- | --- | --- |
| 1 | Control 1 | wt/wt (I) | f | n a | n a | 2009/41 | 57 |
| 2 | Control 2 | wt/wt (II) | f | n a | n a | 2007/25 | 56 |
| 3 | Control 3 | wt/wt (III) | f | n a | n a | 2014/60 | 53 |
| 4 | Control 4 | wt/wt (IV) | m | n a | n a | 2014/71 | 53 |
| 5 | ALS | C9orf72 exp/wt | f | 53 | 29 | 2008/55 | 50 |
| 6 | FTD | C9orf72 exp/wt | m | 65 | 81 | 2011/67 | 43 |
| 7 | ALS | SOD1 A4V/wt | m | 62 | 16 | 2005/62 | 25 |
| 8 | ALS | SOD1 H46R/wt | m | 54 | >187\* | 2007/58 | 26 |
| 9 | ALS | SOD1 G127X/wt (I) | m | 34 | >9 | 2007/23 | 24 |
| 10 | ALS | SOD1 E78\_R79insSI/wt | f | 42 | 20 | 2008/43 | 25 |
| 11 | ALS | SOD1 N86S/wt | m | 77 | 24 | 2008/77 | 25 |
| 12 | ALS | SOD1 L117V/wt | f | 36 | >160\* | 2008/39 | 48 |
| 13 | ALS | SOD1 D90A/D90A | f | 52 | >368\* | 2008/72 | 47 |
| 14 | ALS | SOD1 D125Tfs\*24/wt | m | 55 | >151\* | 2008/58 | 26 |
| 15 | ALS | SOD1 G93A/wt | m | 42 | 11\*\* | 2012/43 | 28 |
| 16 | Later ALS | SOD1 G127Gfs\*7/wt (II) | m | 71 | 24 | 2007/68 | 25 |
| 17 | ALS | SOD1 A4V/wt | f | 73 | 10 | 2014/73 | 23 |
| 18 | PBP | FUS Q23L/wt | m | 35 | 175 | 2012/46 | 57 |
| 19 | ALS | TBKI 450Kfs\*14/wt | m | 55 | >90\* | 2014/57 | 59 |
| 20 | Unaffected | TBK A417X/wt | f | asymptomatic | n a | 2015/59 | 57 |
| 21 | ALS | TBK M598V/wt | f | 63 | 56 | 2014/64 | 52 |
| 22 | Unaffected | TBK 690-713del/wt | f | asymptomatic | n a | 2014/45 | 59 |

## Slide 14
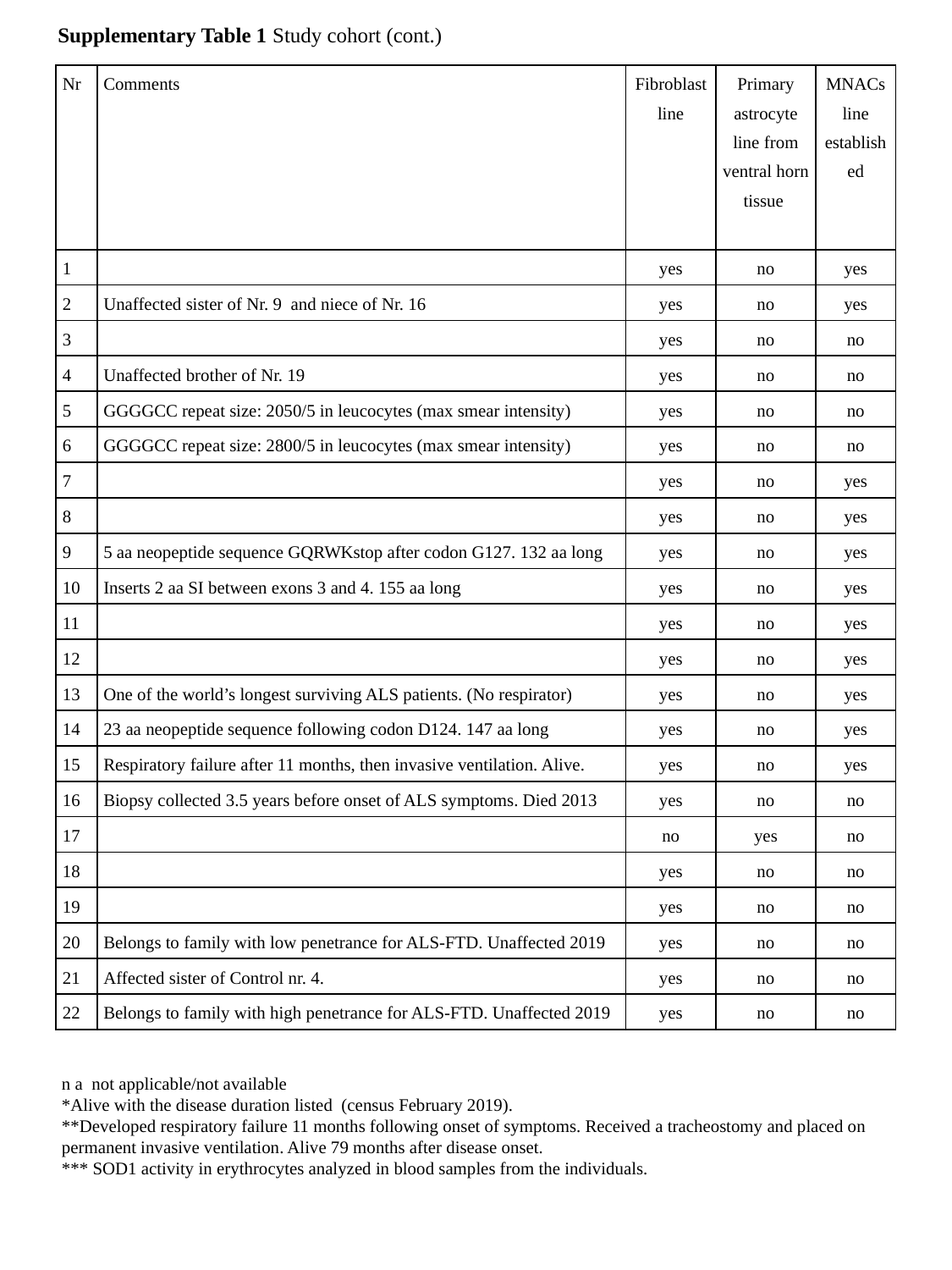

Supplementary Table 1 Study cohort (cont.)
| Nr | Comments | Fibroblast line | Primary astrocyte line from ventral horn tissue | MNACs line established |
| --- | --- | --- | --- | --- |
| 1 | | yes | no | yes |
| 2 | Unaffected sister of Nr. 9 and niece of Nr. 16 | yes | no | yes |
| 3 | | yes | no | no |
| 4 | Unaffected brother of Nr. 19 | yes | no | no |
| 5 | GGGGCC repeat size: 2050/5 in leucocytes (max smear intensity) | yes | no | no |
| 6 | GGGGCC repeat size: 2800/5 in leucocytes (max smear intensity) | yes | no | no |
| 7 | | yes | no | yes |
| 8 | | yes | no | yes |
| 9 | 5 aa neopeptide sequence GQRWKstop after codon G127. 132 aa long | yes | no | yes |
| 10 | Inserts 2 aa SI between exons 3 and 4. 155 aa long | yes | no | yes |
| 11 | | yes | no | yes |
| 12 | | yes | no | yes |
| 13 | One of the world’s longest surviving ALS patients. (No respirator) | yes | no | yes |
| 14 | 23 aa neopeptide sequence following codon D124. 147 aa long | yes | no | yes |
| 15 | Respiratory failure after 11 months, then invasive ventilation. Alive. | yes | no | yes |
| 16 | Biopsy collected 3.5 years before onset of ALS symptoms. Died 2013 | yes | no | no |
| 17 | | no | yes | no |
| 18 | | yes | no | no |
| 19 | | yes | no | no |
| 20 | Belongs to family with low penetrance for ALS-FTD. Unaffected 2019 | yes | no | no |
| 21 | Affected sister of Control nr. 4. | yes | no | no |
| 22 | Belongs to family with high penetrance for ALS-FTD. Unaffected 2019 | yes | no | no |
n a not applicable/not available
*Alive with the disease duration listed (census February 2019).
**Developed respiratory failure 11 months following onset of symptoms. Received a tracheostomy and placed on permanent invasive ventilation. Alive 79 months after disease onset.
*** SOD1 activity in erythrocytes analyzed in blood samples from the individuals.
